# *Latilactobacillus sakei* CNTA 173 Reduces Fat Mass by Modulating Sphingolipid Metabolism in Diet-Induced Obese Wistar Rats

**DOI:** 10.1021/acs.jafc.5c10238

**Published:** 2025-10-30

**Authors:** Ignacio Goyache, Lorena Valdes-Varela, Raquel Virto, Iñigo Clemente-Larramendi, Miguel López-Yoldi, Ana Romo-Hualde, Ana Gloria Gil, Fermín I. Milagro, Paula Aranaz

**Affiliations:** † Faculty of Pharmacy and Nutrition, Department of Nutrition, Food Science and Physiology, 16754 University of Navarra, 31008 Pamplona, Spain; ‡ Center for Nutrition Research, University of Navarra, c/Irunlarrea 1, 31008 Pamplona, Spain; § CNTA, Ctra. NA-134 Km 53, San Adrián 31570, Navarra, Spain; ∥ Department of Pharmacology and Toxicology, University of Navarra, Navarra 31008, Spain; ⊥ Toxicology Unit, Drug Development Unit University of Navarra (DDUNAV), University of Navarra, Navarra 31008, Spain; # Navarra Institute for Health Research (IdiSNA), Pamplona 31008, Spain; ¶ Centro de Investigación Biomédica en Red de la Fisiopatología de la Obesidad y Nutrición (CIBEROBN), Instituto de Salud Carlos III, 28029 Madrid, Spain

**Keywords:** Latilactobacillus sakei, probiotic, Wistar rats, diet-induced obese model

## Abstract

The use of probiotics with health-promoting activities has emerged as a strategy to combat specific hallmarks of obesity, such as excessive fat accumulation and associated inflammation. Here, we investigate the physiological and metabolic effects of *Latilactobacillus sakei* CNTA 173 (10^9^ CFU/day) in diet-induced obese Wistar rats. Biochemical, 16S microbiota, untargeted metabolomic, and gene expression analyses were performed, as well as an in vivo toxicological study to demonstrate its safety. Probiotic-supplemented rats showed reduced adiposity, an improved inflammatory marker profile, and enhanced glucose tolerance, partly by modulating fecal microbiota composition and regulating the production of novel plasma metabolites, specifically sphingolipid-derived mediators such as ceramides. Moreover, no adverse effects were observed in an in vivo toxicity evaluation of this strain in Wistar rats. Our results unveil the fat-reducing and anti-inflammatory effects of *L. sakei* CNTA 173 and point out the potential use of this probiotic for the prevention of obesity-related disturbances.

## Graphical abstract

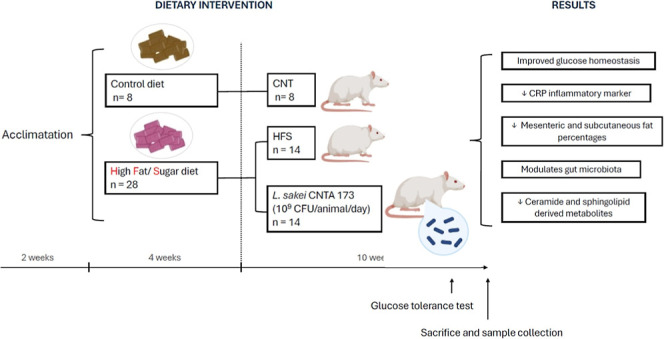

## 1 Introduction

Obesity is a chronic multifactorial disease characterized by a high body fat percentage and constitutes one of the greatest global health challenges.^1^ Closely linked to obesity, cardiovascular diseases, type-2 diabetes, dyslipidaemia, and metabolic dysfunction-associated steatotic liver disease (MASLD) are also experiencing an alarming growth rate.^2,3^

In recent years, the human gut microbiome has become an important target to consider in the prevention and development of obesity due to the influence that intestinal microorganisms exert on human metabolic health. Proposed mechanisms by which the gut microbiome may influence obesity include increased energy harvest from the diet, chronic low-grade endotoxemia, regulation of biologically active fatty acid (FA) composition, and modulation of gut-derived peptide secretion. For this reason, there is a growing interest in therapies that can modulate obesity-related dysbiosis, including probiotic interventions.^4–7^

The great diversity of bacterial strains among the ancient genus *Lactobacillus* calls for the discovery of new strains that could potentially have probiotic properties.^8^ One species that has been previously reported to have antiobesity properties in animal models is *Latilactobacillus sakei*. Thus, the administration of *L. sakei* WIKIM31 reduced body weight gain, epididymal fat mass, triglyceride, and total cholesterol levels in diet-induced obese (DIO) mice.^9^ In the same line, we recently described *L. sakei* CNTA 173 as a novel, promising probiotic candidate with antiobesity and health-promoting effects in *Caenorhabditis elegans*.^10^ However, the specific effects of *L. sakei* strains in mammalian models are largely unexplored, together with the specific mechanisms underlying these effects. Moreover, before considering its use in humans, safety studies in rodent models are crucial for evaluating the tolerability and systemic effects of a specific probiotic candidate, to ensure not only its effectiveness but also its safety for further clinical development.

Here, we investigate the antiobesogenic, anti-inflammatory, and normoglycemic potential of the probiotic *L. sakei* CNTA 173 strain in a DIO Wistar rat model, together with its potential mechanisms of action through 16S and untargeted metabolomic analyses. The functional study is accompanied by an in vivo toxicological evaluation to demonstrate its safety.

## 2 Material and Methods

### 2.1 Bacterial Strain and Growth Conditions

The strain used in this study was *Latilactobacillus sakei* CNTA 173, which was collected from pea byproducts and derived from CNTA culture collection (San Adrián, Navarra, Spain).^10^ The strain was grown in MRS broth (Merck) at 30 °C under anaerobic conditions. The bacterial stock, kept at −80 °C in a cryovial (Scharlab), was spread onto the surface of agar-MRS and incubated for 2 days. A single colony was picked to inoculate 10 mL MRS broth, which, after 19 h incubation, was used to inoculate bottles of 800 mL fresh MRS broth (10^6^ CFU/mL). The bottles containing inoculated media were incubated under anaerobic conditions at 30 °C for 14 h. The cells were harvested from 4 L of a 14 h culture of *L. sakei* CNTA 173 grown in MRS medium via centrifugation (4347*g*, 10 min, 4 °C). The cell pellet was washed once with Buffered Peptone Water and resuspended in 80 mL of cryoprotectant solution (20% skim milk and 5% sucrose). The resulting biomass was frozen at −80 °C for 24 h and lyophilized using a LyoMicron laboratory freeze-dryer (Cool vacuum Technologies, S.L., Granollers, Spain).

### 2.2 DIO Wistar Rats

Three-week-old male Wistar rats (*n* = 36) were obtained from Envigo (Envigo Research Models and Services, Indianapolis, IN). Rats were kept in an isolated room with controlled temperature (21 to 23 °C) and humidity (50% ± 10%) and a 12 h:12 h artificial light/dark cycle. Rats were acclimatized to the experimental facility for 1 week prior to the experiment with a control diet (2014, Teklad, Global 14% Protein Rodent Maintenance Diet). At the beginning of the study, 28 out of 36 rats started receiving a high fat/high sucrose diet (D12451, Research Diets Inc., New Brunswick, NJ) for 3 weeks to induce weight gain, while the remaining 8 rats (CNT) were maintained on the control diet (2014, Teklad Global 14% Protein Rodent Maintenance Diet) during the whole experiment. After the weight gaining period, the HFS-fed rats were divided into two groups (*n* = 14): nonsupplemented HFS group (HFS) and *L. sakei*-supplemented HFS group (*L. sakei*), orally supplemented with 10^9^ CFUs per animal and day. The probiotic was administered mixed with the diet in a formulation that was prepared every 3 days. The supplementation was maintained for 10 weeks, with rats being housed individually with ad libitum water. Food intake was adjusted to ensure the optimal intake of the supplementation, while food intake differences were avoided among HFS groups. At week 10, all rats were euthanized by decapitation, and trunk blood was collected to obtain serum and plasma samples for subsequent biochemical analyses. Tissues, including liver, kidneys, spleen, gastrocnemius muscle, brown adipose tissue, and white adipose tissue (WAT) depots (mesenteric, retroperitoneal, epididymal, and subcutaneous), were extracted, weighed, and stored at −80 °C. All animal procedures were performed in accordance with the national and institutional Guidelines for Care and Use of Laboratory Animals, with the consent of the Food Safety and Environmental Health Service of the Government of Navarra, Spain. The protocol was approved by the Ethics Committee for Animal Experimentation of the University of Navarra (protocol 038-21E1, approved on 25th February 2022).

### 2.3 Biochemical Analyses

Serum total cholesterol, HDL-cholesterol, triacylglycerides (TAG), glucose, aspartate transaminase (AST), and alanine transaminase (ALT) were quantified with specific kits adapted for the Pentra C200 analyzer (HORIBA ABX, Montpellier, France). Specific ELISA kits (Life Technologies, Grand Island, NY) were used to quantify plasma concentrations of C-reactive protein (CRP) and monocyte chemotactic protein-1 (MCP-1). Serum insulin was also quantified with a specific ELISA kit (Mercodia AB, Uppsala, Sweden). Insulin resistance was evaluated by the homeostasis model of insulin resistance (HOMA-IR) formula [serum glucose levels (mmol L^–1^) × insulin levels (mU L^–1^)]/22.5. The Triglyceride-Glucose (TyG) index was calculated by the formula: ln [triglycerides (mg/dL) × glucose (mg/dL)/2]. The Atherogenic index of plasma (AIP) was calculated by the formula AIP = log­[triglycerides (mg/dL)/HDL-cholesterol (mg/dL)].

### 2.42.4 Intraperitoneal Glucose Tolerance Test

An IGTT was performed at week 9 of the study (the week before the sacrifice). Food was removed 15 h before the test, leaving the animals with access to only water. Before the test, rats were weighed, and d-glucose (1 g kg^–1^) was intraperitoneally administered. Glycaemia was quantified using a glucometer and blood glucose test strips (Optium Plus, Abbott Diabetes Care, Witney, Oxon, UK) by venous tail puncture before (baseline) and after the glucose administration (at min 20, 40, 60, 90, and 150). Glucose content (mg dL^–1^) was used to calculate the area under the curve (AUC) by using the following formula previously published.^11^

### 2.5 Determination of Hepatic Triglyceride Content

A 100 mg sample was taken from the same lobe of each animals liver, dissolved in ethanol with 10% potassium hydroxide, and incubated overnight at 55 °C with gentle shaking. Each sample was then centrifuged for 5 min at 1200 rpm, and the supernatants were collected, diluted in 50% (v/v) ethanol, and mixed in a vortex. Samples were then mixed in a 1:1 ratio with 1 M magnesium chloride, incubated for 10 min on ice, and centrifuged for 5 min at 1200 rpm. The triglyceride contents of these samples were quantified using the HK-CP kit adapted for the Pentra C200 analyzer (HORIBA ABX, Montpellier, France).

### 2.6 RNA Extraction and Quantitative Polymerase Chain Reaction Analyses

RNA was extracted from mesenteric fat and liver samples, and qPCR analyses were carried out as reported previously.^12^ Briefly, 500 mg of mesenteric fat and liver tissues from each animal were treated with TRIzol RNA isolation reagent (Thermo-Fisher Scientific Inc., Paisley, UK) to extract total RNA. The concentration and purity of RNA were determined at 260/280 nm using a NanoDrop ND-1000 spectrophotometer (Thermo Fisher Scientific, Wilmington, DE). Subsequently, 500 ng of RNA was treated with DNase I (DNase I-RNase free, Invitrogen Life Technologies, Paisley, UK) according to the standard protocol and was reverse-transcribed using 200 IU of M-MLV-RT (Invitrogen Life Technologies, Paisley, UK) in the presence of 40 IU of recombinant RNAsin ribonuclease inhibitor (Promega, Madison, WI), with an incubation of 10 min at 25 °C, 50 min at 37 °C and 15 min at 70 °C.

Gene expression analyses were performed by quantitative-real-time PCR (qPCR) in triplicate using the TaqMan Universal PCR master mix with specific probes (Table S1) from Applied Biosystems (TaqMan Gene Expression Assays) and Integrated DNA Technologies (Integrated DNA Technologies Inc., Coralville, IA, using a CFX384 Touch Real-Time PCR Detection System (Bio-Rad Laboratories, Hercules, CA). The expression levels of each gene were normalized using the expression of TATA box binding protein (*Tbp*) as a reference housekeeping gene. Gene expression differences between *SAKEI*-treated and untreated samples were quantified using the relative quantification 2^–ΔΔCt^ method.^13^

### 2.7 Fecal Sample Collection and 16S Analyses

At week 10 of the supplementation, fresh fecal samples were collected from each animal, ensuring their precedence, and stored at −80 °C. The following bacterial DNA isolation and sequencing analysis were performed by the CimaLab diagnostic genomics unit (University of Navarra, Pamplona, Spain). The comparison with a curated Illumina database allowed for a characterization of the sequenced V3–V4 16S genetic regions, grouped in operational taxonomic units and a classification at phylum, class, order, family, genus, or species level of the genomic data. The rarefaction curve was first calculated, and then linear discriminant analysis effect size (LefSe) analysis was performed to compare the relative abundance of *L. sakei* CNTA 173 among the three experimental groups, with a statistical p-value cutoff set at 0.05. Finally, the relative abundance of each taxon at different taxonomical levels was compared between the HFS and *L. sakei* groups using the Linear Model comparison, with a 0.05 adjusted p-value cutoff (available at http://www.microbiomeanalyst.ca).

### 2.8 Untargeted Metabolomics Analysis

Serum samples were taken to perform untargeted metabolomics as described previously.^14^ Briefly, 150 μL of serum aliquot from each animal were vortexed and diluted with 450 μL of methanol (MeOH), mixed, and centrifuged for 10 min at 7280*g*. Samples were processed and then analyzed in an Agilent Technologies 1200 liquid chromatographic system equipped with a 6220 Accurate-Mass TOF LC/MS, operated in positive electrospray ionization mode (ESI^+^) and negative mode (ESI^–^) controlled using MassHunter Workstation 06.00 software (Agilent Technologies, Santa Clara, CA). Total injection volume was 15 μL per sample, and the flow rate was 0.5 mL min^–1^. ESI conditions were as follows: gas temperature, 350 °C; drying gas, 10 L min^–1^; nebulizer, 45 psig; capillary voltage, 3500 V; fragmentor, 175 V; and skimmer, 65 V. The instrument was set out to acquire over the *m*/*z* range of 100–2000 with an acquisition rate of 1.03 spectra/s.

Liquid chromatography–mass spectrometry data were analyzed using XCMS online software (https://xcmsonline.scripps.edu) to detect and align features.^14^ The alignment used a 0.2 min retention time and a 0.002 Da mass tolerance window. Metaboanalyst software (https://www.metaboanalyst.ca/) was used to identify those metabolites statistically significant between HFS and *L. sakei* groups, based on the Volcano plot (*p* < 0.001, fold change = 4.5) and Partial Least Squares Discriminant Analysis (PLS-DA) analysis (VIP > 1.03). A first attempt was made to identify metabolites using METLIN (https://metlin.scripps.edu/index.php) within a mass accuracy below 5 mDa, the scientific literature, and the metabolic pathways reported in the Kyoto Encyclopaedia of Genes and Genomes (KEGG) database (http://www.genome.jp/kegg/), the Human Metabolome Database (HMDB) (http://www.hmdb.ca/), and Lipid maps (http://www.lipidmaps.org/).

### 2.9 Safety Evaluation of *L. sakei* CNTA 173 Administration in Wistar Rats

Oral administration of *L. sakei* CNTA 173 (10^9^ CFU/animal/day) was evaluated for possible toxic adverse effects by conducting a repeated dose assay of 10 weeks. The in vivo toxicological experiment was approved by the Ethics Committee on Animal Experimentation of the University of Navarra (protocol GLP like DDUNAV 012/22). Wistar rats (*n* = 20, 10 male and 10 female) were purchased from Envigo (Envigo Research Models and Services, Barcelona, Spain). Rats were identified and distributed in four groups: control diet female rats (CNT-F, *n* = 5), control diet male rats (CNT-M, *n* = 5), *SAKEI* female rats (*SAKEI*-F, *n* = 5), and *SAKEI* male rats (*SAKEI*-M, *n* = 5). Animals were housed in Makrolon cages in groups of 5 animals per cage. After an acclimation period to the environmental conditions (12 h day/night cycle, temperature 22 ± 2 °C, relative humidity 50 ± 10%, and water *ad libitum*), all animals consumed the control diet (2014, Teklad Global 14% Protein Rodent Maintenance Diet) during the whole experiment. *SAKEI* groups received daily 10^9^ CFUs of *L. sakei* CNTA 173 embedded in a dense paste obtained after mixing the powder diet with water, while the HFS group received only the vehicle. Food intake was evaluated weekly, and *ad libitum* water was used, 7 days a week, for 10 weeks.

The study included a general evaluation of viability/mortality, food consumption, and general symptoms. A total of two Irwin complete tests were carried out at the beginning of the study (basal) and the week prior to the sacrifice. A more in-depth study of serum biochemical and hematological parameters was performed the day before the sacrifice using a Cobas c111 analyzer (Hoffmann-La Roche; Basel, Switzerland) and a Sysmex XT-1800i apparatus (Roche 2011, Kobe, Japan), respectively. The day before the sacrifice, animals were individualized and kept in metabolic cages to determine daily volume, appearance, color, and smell of the urine, together with an evaluation of biochemical parameters using a Cobas u411 analyzer (Hoffmann-La Roche; Basel, Switzerland). After sacrifice, all animals were subjected to a macroscopic anatomopathological study, including general state, detection of wounds or external abnormalities, an “in situ” evaluation of the organs, and an individualized exam of each extracted organ. From each animal, the following organs were extracted: spleen, heart, adrenal glands, liver, pancreas, kidneys, testicles, thyme, and the whole gastrointestinal tract. Histological samples were stored in 4% formaldehyde except testicles, which were fixated in Davidsońs fixing solution and preserved in 70% ethanol after 48 h. All samples were sent to Patconsult.LAB.SL (Barcelona, Spain) for further processing, macroscopic and microscopic evaluation.

### 2.10 Statistical Analyses

All statistical analyses were performed using StataSE v14 software (StataCorp LLC, College Station, TX and graphed using GraphPad Prism 9.0 (GraphPad Software, San Diego, CA). Data are presented as the mean ± the standard deviation (SD). One-way ANOVA was used to assess differences between the control, HFS, and *SAKEI* groups, followed by uncorrected Fisher’s LSD post hoc test for multiple comparisons. In the 16S sequencing analyses, ANOVA followed by Welch’s *t*-test was used for alpha diversity indexes. In the 16S and metabolomic analyses, multivariate models such as linear discriminant analysis effect size (LEfSe), principal component analysis (PCA), PLS-DA, and compound Poisson lognormal model (CPLM) were applied through MicrobiomeAnalyst and MetaboAnalyst platforms to compare the studied variables. Correlation analyses were conducted to find associations between microbial taxa relative abundances, metabolite levels, and physiological parameters. A *p*-value <0.05 was considered statistically significant.

## 3 Results

### 3.1 *L. sakei* CNTA 173 Administration Lowers WAT by Regulating Adipogenesis Key Genes

During the three-week weight gain period, animals on the high-fat-sucrose diet showed significantly greater weight gain compared to those fed the control or standard diet (CNT). As a result, the CNT group exhibited a lower initial body weight at the beginning of supplementation than the other two groups, with no significant differences between animals supplemented with *L. sakei* CNTA 173 (*SAKEI*) and those not supplemented (HFS) (Table 1). After 10 weeks of supplementation, no differences were observed in body weight or weight gain between the SAKEI and HFS groups. Additionally, the probiotic did not affect the diet intake during the experiment.

**Table 1:** Caloric Intake and Body Weight Measurements of the Control (CNT), HFS, and *L. sakei* CNTA 173-Treated (*SAKEI*) Groups^a^ ^b^

	CNT (*n* = 8)	HFS (*n* = 14)	SAKEI (*n* = 14)
initial body weight (*g*)	340.0 ± 6.8 *	362.6 ± 6.9	362.9 ± 6.9
body weight gain (Δ*g*)	101.2 ± 4.5 ***	146.5 ± 2.6	143.0 ± 8.7
diet intake (kcal day^–1^)	AL	102.1 ± 0.8	104.2 ± 3.3

a One-way ANOVA statistical analysis was performed, followed by Fisher’s LSD test multiple comparison (*p < 0.05; ***p < 0.001). Data are expressed as the mean ± SEM.

b AL: ad libitum.

No significant differences were found between groups upon comparison of the organ weights (Table S2). In the case of WAT depots, the CNT group exhibited significantly reduced weight of epididymal, retroperitoneal, mesenteric, and subcutaneous fats (Figure 1), demonstrating the obesogenic effect of the HFS diet on adiposity. Regarding HFS-fed groups, no differences were observed in epididymal (Figure 1A) or retroperitoneal (Figure 1B) fat depots between *SAKEI* and HFS. However, despite no differences having been shown in the total body weight, animals supplemented with the probiotic exhibited a lower percentage of mesenteric (Figure 3C) and subcutaneous (Figure 3D) fat depots, in comparison with the nonsupplemented HFS rats.

**Figure 1:**
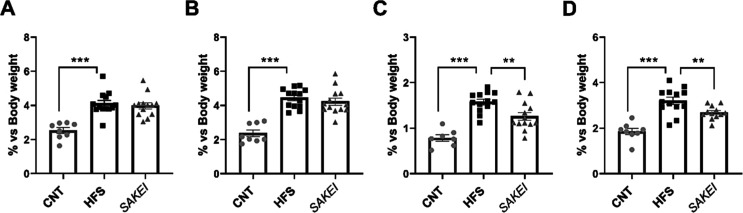
Weight percentage of isolated fat deposits. (A) Epididymal fat. (B) Retroperitoneal fat, (C) mesenteric fat, and (D) subcutaneous fat. One-way ANOVA statistical analysis was performed, followed by uncorrected Fisher’s LSD test for multiple comparisons (**p < 0.01; ***p < 0.001). Data are expressed as the mean ± SEM, with a scatter dot plot representing individual values.

We then analyzed the expression of key genes related to lipid and glucose metabolism, including those involved in signaling regulation, glucose utilization, and lipid biosynthesis and oxidation, in mesenteric fat samples via qPCR. As shown in Figure 2, there was a normalization in the expression of both *Lep* (Figure 2A) and *Adipoq* (Figure 2B) genes in the *SAKEI* group, which were significantly lower when compared to the HFS group and equal to the expression in the CNT group. In the case of genes involved in FA biosynthesis, the probiotic group suffered a significant downregulation of *Pparg* (Figure 2C), *Srebf1* (Figure 2D), *Fabp4* (Figure 2E), and *Plin* (Figure 2F), while *Pgc1a* (or *Ppargc1a*), a mitochondrial regulator of FA oxidation and glucose uptake, was significantly upregulated (Figure 2G) by the probiotic. In this line, WAT expression levels of different glucose metabolism-related genes, including *Slc2a4* (Figure 2H), *InsR* (Figure 2I), and *Pdk4* (Figure 2J) were down-regulated in SAKEI samples, in comparison with the untreated HFS group.

**Figure 2:**
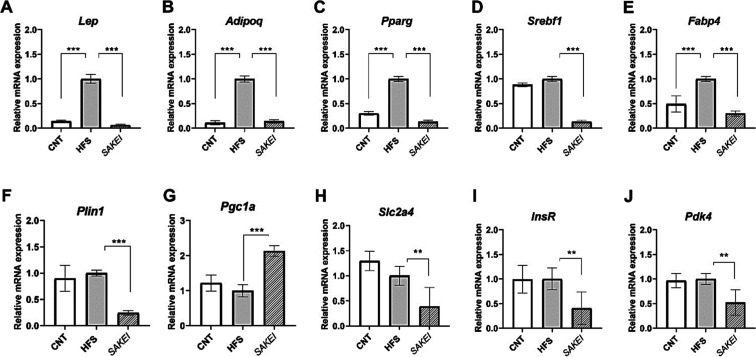
Gene expression analyses in mesenteric adipose tissue quantified by real-time PCR (qPCR). Gene expression levels (Mean ± SEM) were normalized to the housekeeping gene (*Tbp*). (A) *Lep* gene expression. (B) *Adipoq* gene expression. (C) *Pparg* gene expression. (D) *Srebf1* gene expression. (E) *Fabp4* gene expression. (F) *Plin1* gene expression. (G) *Pgc1a* gene expression. (H) *Slc2a4* gene expression. (I) *InsR* gene expression. (J) *Pdk4* gene expression. Data are expressed using the 2^–ΔΔCt^ method. A one-way ANOVA followed by LSD́s Fisher to evaluate statistical differences between groups (**p < 0.01; ***p < 0.001).

Gathering these results together, treatment with *L. sakei* CNTA 173 led to a lower accumulation of mesenteric and subcutaneous fat depots in the DIO Wistar rats by promoting the downregulation of genes associated with adipokine signaling, FA biosynthesis, and enhancing FA oxidation. This reprogramming of visceral adipose tissue is further supported by the significant down-regulation of glucose uptake- and gluconeogenesis-related genes in mesenteric adipocytes from the SAKEI group. Altogether, these molecular changes bring the SAKEI group closer to the CNT, indicating its potential to mitigate HFS diet-induced metabolic dysregulation at the level of visceral adiposity.

### 3.2 Supplementation with *L. sakei* CNTA 173 Improves Glucose Homeostasis and Reduces Inflammation

A week previous to the sacrifice, we performed an IGTT, where we observed that the control diet-fed animals (CNT) exhibited significantly reduced levels in the glycaemia at the times 20, 40, and 60 min, in comparison with the HFS diet-fed groups (Figure 3A). This improvement of glucose homeostasis was also evidenced by a significant reduction in AUC (Figure 3B), in comparison with the HFS group. Regarding the probiotic effect, although SAKEI-treated rats did not exhibit differences in the total AUC, these animals showed significantly lower plasma glucose levels at 20 and 40 min after glucose administration, in comparison with nonsupplemented HFS animals, suggesting a normoglycemic activity of *L. sakei* CNTA 173 in this model.

**Figure 3:**
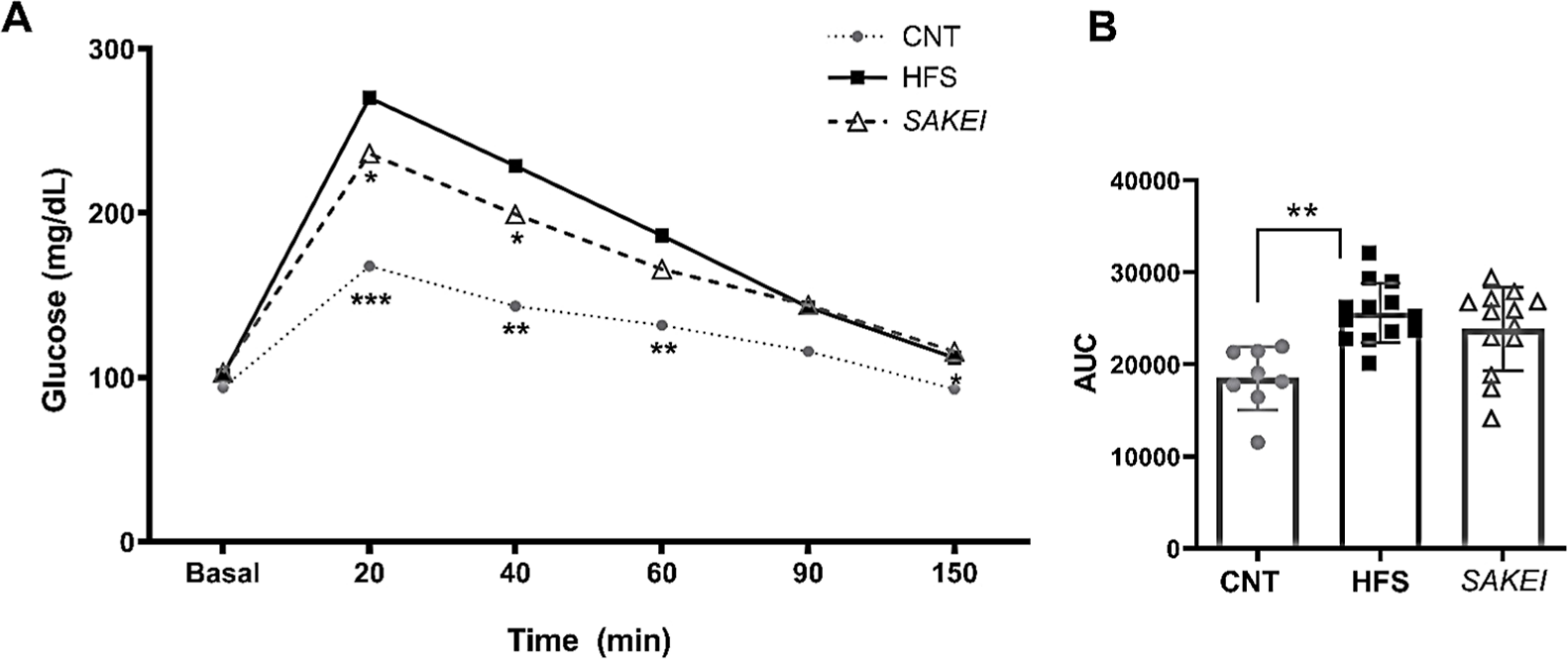
*L. sakei* CNTA 173 improves glucose tolerance in obese rats after an IGTT. (A) Plasma glucose levels over the 150 min-IGTT represented as the individual measures taken at the shown times. (B) AUC of the glucose tolerance curve. Differences were studied using one-way ANOVA statistical analysis, followed by uncorrected Fisher’s LSD for multiple comparisons (*p < 0.05; **p < 0.01; ***p < 0.001).

After 10 weeks of probiotic supplementation, animals were euthanized, and serum samples were collected to perform biochemical analysis. As shown in Table S3, the *SAKEI* group did not significantly induce differences in any of the analyzed parameters, including cholesterol and glucose metabolism-related markers. However, it is remarkable that no differences were also observed between the CNT and the HFS groups in the circulating levels of cholesterol, ALT, AST, triglycerides, or insulin. Only the HDL-cholesterol levels were significantly different between the CNT and the HFS groups. This lack of effect on biochemical parameters suggests that consumption of the HFS diet failed to induce dyslipidaemia or hyperglycaemia in our Wistar rat obesity model. Thus, although a change in the HOMA insulin resistance index calculation is perceived (Figure 4A), these differences do not reach statistical significance compared with the CNT group. Calculation of the TyG index (Figure 4B) and the atherogenic index of plasma (AIP, Figure 4C) confirms this fact. However, the analysis of the intrahepatic triglyceride levels demonstrated the effect of the HFS diet on liver steatosis, without differences between probiotic- and nonsupplemented HFS groups (Figure 4D).

**Figure 4:**
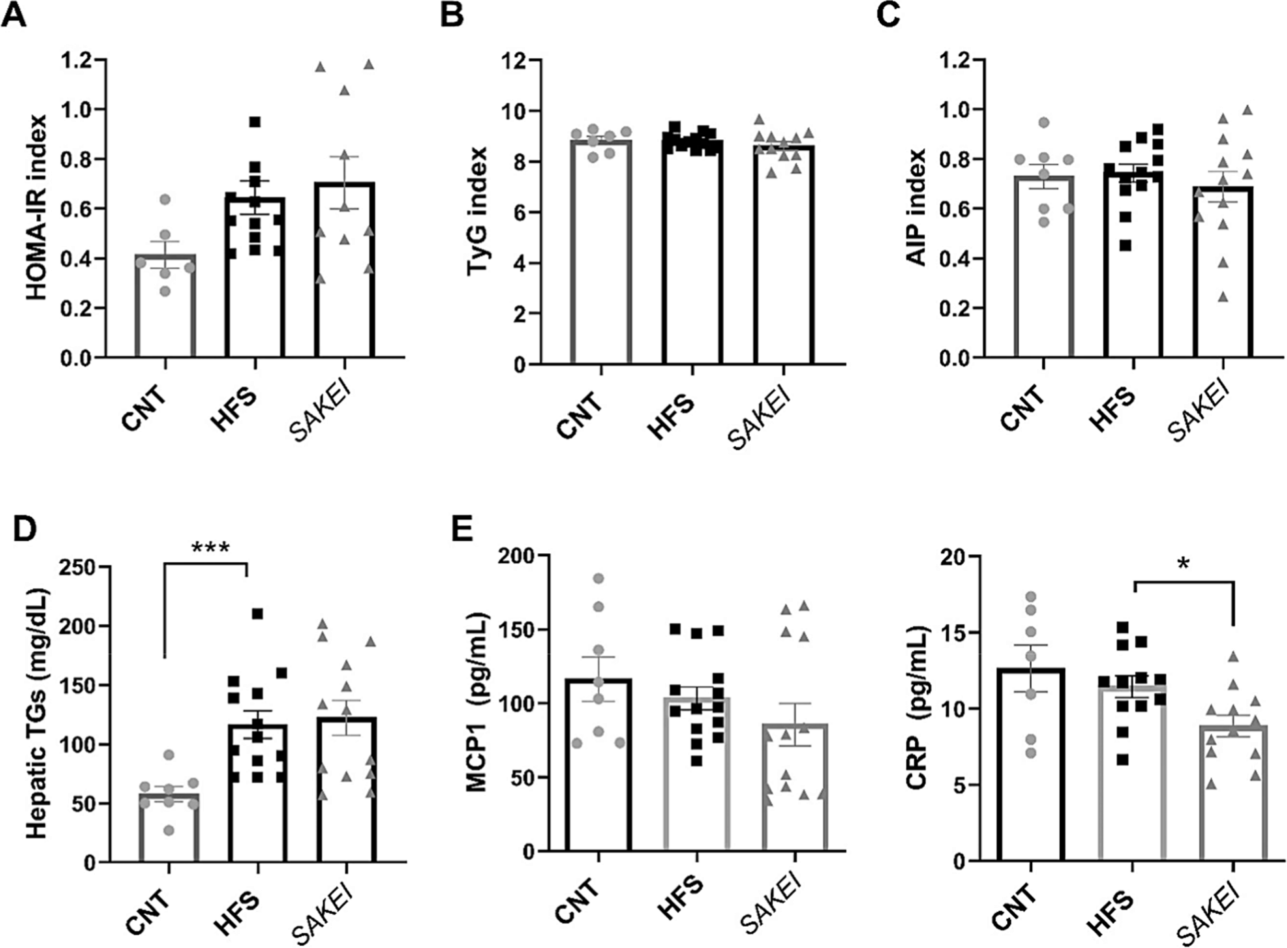
Representation of the HOMA-IR (A), TyG (B), and AIP (C) indexes. (D) Levels of intrahepatic triglycerides among groups. (E) MCP-1 plasma levels represented for each individual and separated into the three experimental groups. (F) CRP plasma levels represented for each individual and separated into the three experimental groups. Data are expressed as the mean ± SEM. Statistical differences between groups were studied using one-way ANOVA statistical analysis followed by uncorrected Fisher’s LSD test for multiple comparisons (*p < 0.05; ***p < 0.001).

Finally, we investigated the potential anti-inflammatory activity of *L. sakei* CNTA 173 through the quantification of pro-inflammatory cytokines in plasma samples. Although no differences were reached in the levels of MCP-1 (Figure 4E) between the probiotic and nonsupplemented HFS animals, supplementation with *L. sakei* CNTA 173 significantly reduced the circulating levels of the pro-inflammatory marker CRP (Figure 4F). No significant differences were observed between the HFS and CNT groups.

In summary, our results suggest that, despite not having achieved the model of insulin resistance and dyslipidemia induced by the HFS diet, supplementation with *L. sakei* CNTA 173 during 10 weeks exerts health promoting effects by improving glucose tolerance and reducing glycemic peaks at specific time points, together with ameliorating the low-grade inflammatory state by reducing CRP levels.

### 3.3 16S Analysis Reveals that *L. sakei* CNTA 173 Abundance Is Correlated with the Health-Promoting Activities

The day previous to the sacrifice, fresh fecal samples were obtained from each animal of the experiment, and 16S sequencing was performed. First, an analysis of the samples within the groups was initiated by visualizing the rarefaction curve of the samples within each group (Figure S1). Therefore, the samples −02, −96 (CNT), −25, −26 (HFS), and −48 (*SAKEI* group) were discarded for posterior analysis. Also, the analyzed samples from the CNT group had a much lower read count than the other two groups. After discarding the mentioned samples, we performed an overview of the influence that both the HFS diet and the probiotic supplementation induced on alpha diversity. As shown in Figure S2, a significantly lower Shannon index was observed in the CNT group when compared to the HFS and *SAKEI* groups, with no significant differences between them. The Simpson index demonstrated that the only significant difference in the alpha diversity was found between the CNT and HFS groups.

Following this overall assessment of the samples and having demonstrated the different microbiota composition of the CNT group compared to the rest of the groups due to the different compositions of the diet, the subsequent analysis was performed only comparing the HFS and *SAKEI* groups. As shown in Figure 5, we found significant differences in the abundances of two families: Ruminococcaceae (Figure 5A), which was relatively less abundant in the probiotic group, and Carnobacteriaceae, with an opposite distribution (Figure 5B). Following with the taxonomical classification, only the *Isobaculum* genus was significantly more abundant in the *SAKEI*-treated group in comparison with the HFS (Figure 5C). Lastly, we found that the species *Isobaculum melis* (Figure 5D) and *Latilactobacillus sakei* were significantly more abundant in the *SAKEI*-treated group, with the last one being present only in the treated group.

**Figure 5:**
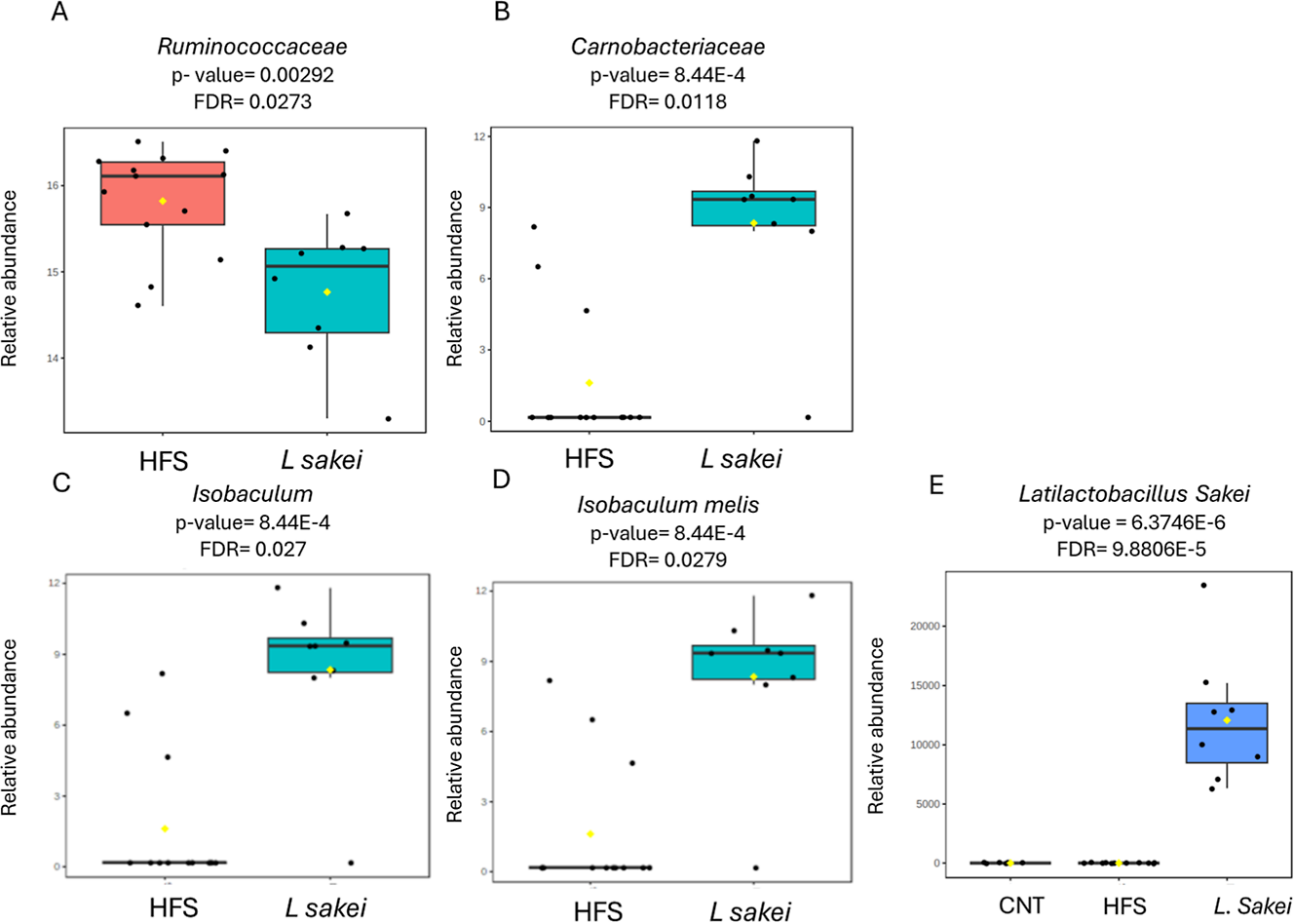
16S sequencing study. (A,B) Statistically significant differences in abundance rates of families, (C) genera and (D,E) species. 16S statistical analyses and the abundance of the bacteria were calculated by a Multiple Linear Regression with Covariate Adjustment, CPLM, available in the MicrobiomeAnalyst software. Relative abundances are represented as the log-transformed-counts.

This result allowed us to confirm the presence of *L. sakei* genetic material in the feces of the treated group, which suggests that the physiological effects observed in the treated group could be related to the presence of this probiotic in the intestine of these animals. To investigate this, we conducted a correlation analysis comparing different physiological values with the relative abundance of the probiotic in each animal from the HFS and *SAKEI* groups. We found that the abundance of *L. sakei* identified in the supplemented group exhibited significant negative correlations with the total body weight (Figure 6A), subcutaneous fat percentage (Figure 6B), and CRP circulating levels (Figure 6C) of the animals.

**Figure 6:**
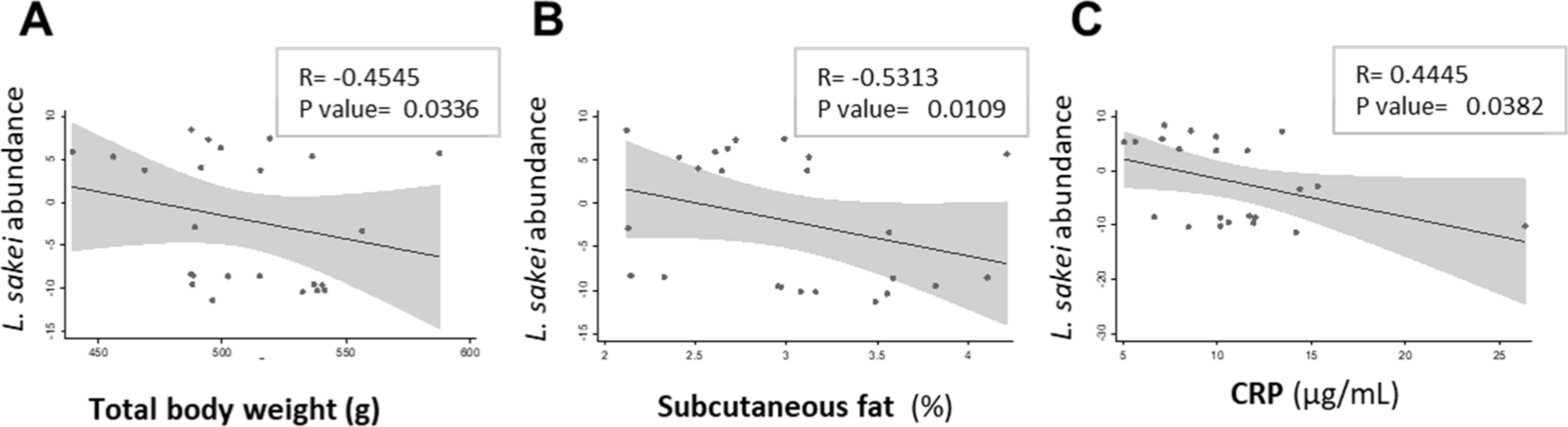
*L. sakei* 173 abundance correlates with physiological parameters. (A) Total weight, (B) subcutaneous fat, and (C) CRP. The Pearson correlation test was performed.

Gathering these results, we have demonstrated the presence of the probiotic DNA in the feces of the supplemented group, together with its gut microbiota modulating activity, including the relative increment of *Isobaculum melis* species. Moreover, the abundance of *L. sakei* CNTA 173 correlates with specific physiological improvements induced by the probiotic, including a reduction in adiposity and inflammation.

### 3.4 Untargeted Metabolomics Reveals Modifications in Sphingolipid Metabolism after Supplementation with *L. sakei* CNTA 173

We performed untargeted metabolomic analyses from serum samples to detect significant differences in the concentrations of specific serum-circulating metabolites that could be attributed to the probiotic activity. In this case, only the HFS and *SAKEI* groups were included. Principal component analyse (PCA) analysis of serum metabolome evidenced the differentiation between the HFS and *SAKEI* samples, in both negative and positive polarities (Figure S3). This result suggests the possible presence of *L. sakei* CNTA 173 circulating metabolites or the inhibition of the production of others; specific features were interrogated in both negative and positive ionization modes. 51 features in the positive ionization mode and 49 in the negative ionization mode were differentially present in treated and untreated HFS animals, after applying predetermined cutoff values (Fold change: 4.5; *p*-value: 0.001; VIP > 1.03). Despite the number of potential features identified, after the comparative analysis with the Metlin database, 69 of the selected candidates presented masses not compatible with any metabolite identified to date, so their identification was not possible, and they were listed as *not found* and finally discarded.

Selecting those features for which potential metabolites had been identified in the *Metlin* database, a correlation analysis was performed between differential metabolites and changes in the microbiota using the Microbiome Analyst tool. This analysis revealed a strong negative correlation between six potential metabolites and the abundance of *L. sakei* CNTA 173 in fecal samples. Moreover, although with reduced correlation levels, these six metabolites were also negatively correlated with the levels of *Isobaculum melis*, the only bacterial species significantly more abundant in the probiotic group (Figure 7A).

**Figure 7:**
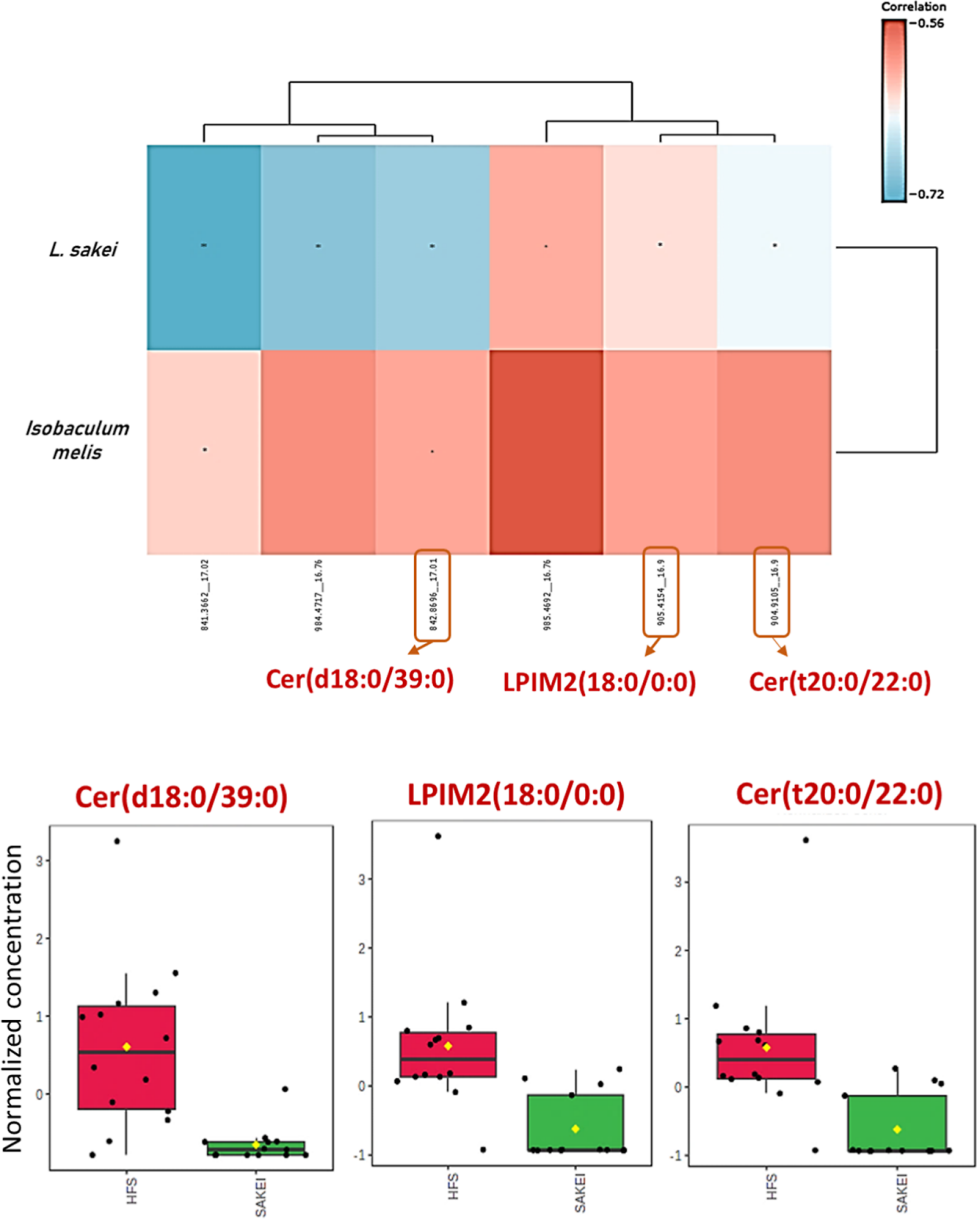
*L. sakei* 173 induces metabolic changes that correlate to its relative abundance in DIO Wistar rat fecal samples. (A) Correlation study of the relative abundance of *L. sakei* and *Isobaculum melis* against six of the most relevant and recognized metabolites after the comparative analysis with Metlin database. (B) Box plot of relative abundance of the metabolites Cer­(d18:0/39:0), LPIM2­(18:0/0:0), and (Cer (t20:0/22:0), respectively, in the HFS and *SAKEI*-treated groups.

Three of the six metabolites, identified as *N*-(nonatriacontanoyl)-sphinganine (Cer­(d18:0/39:0)), 2′-O-(alpha-D-Manp)-6′-O-(alpha-D-Manp)-(1-octadecanoyl-*sn*-glycero-3-phospho-1′-myo-inositol) (LPIM2­(18:0/0:0)) and *N*-(nonatriacontanoyl)-4R-hydroxyeicosasphinganine (Cer (t20:0/22:0)), were significantly more abundant in the HFS group than in the *L. sakei* CNTA 173 (Figure 7B), all are related to the sphingolipid and ceramides metabolism.

To demonstrate the involvement of sphingolipid metabolism in the probiotic mechanism of action, we investigated the expression of some key genes involved in ceramide synthesis and elongation. The gene expression analyses demonstrated that mesenteric fat samples of the *SAKEI* group showed a significant down-regulation of *Cers2* (Figure 8A), *Cers5* (Figure 8B), *Cert1* (Figure 8C), *Sgpl1* (Figure 8D), and *Sphk1* (Figure 8E).

**Figure 8:**
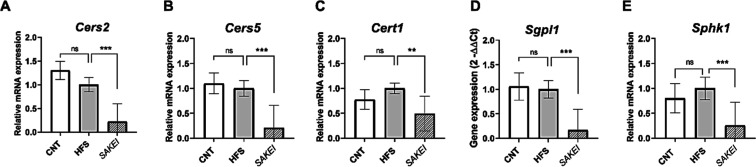
Gene expression analyses in mesenteric adipose tissue quantified by real-time PCR (qPCR). Gene expression levels (Mean ± SEM) were normalized to the housekeeping gene (*Tbp*). Data are expressed using the 2^–ΔΔCt^ method. A one-way ANOVA followed by LSDs Fisher to evaluate statistical differences between groups (ns, not significant; **p < 0.01; ***p < 0.001).

Furthermore, due to the important role that this organ plays in the synthesis and degradation of sphingolipids, including ceramide and sphingosine, and the impact of their dysregulation on the hepatic metabolic health, we also investigated the expression of these genes in the liver from the three groups of the study. These analyses demonstrated that the SAKEI group experienced a significant reduction in the hepatic gene expression of ceramide synthesis-related genes *Cers2* and *Cers5* (Figure 9 A,B), while showing a tendency to increase the *Sgpl1* expression (Figure 9D). No expression was found for *Sphk1* in the liver samples. Moreover, these results were accompanied by the significant reduction of the insulin signaling-related genes *Gsk3b* (Figure 9E), *Pdk4* (Figure 9F), *Insr* (Figure 9F), and *Akt1* (Figure 9H) in the SAKEI-supplemented liver samples, in comparison with the HFS.

**Figure 9:**
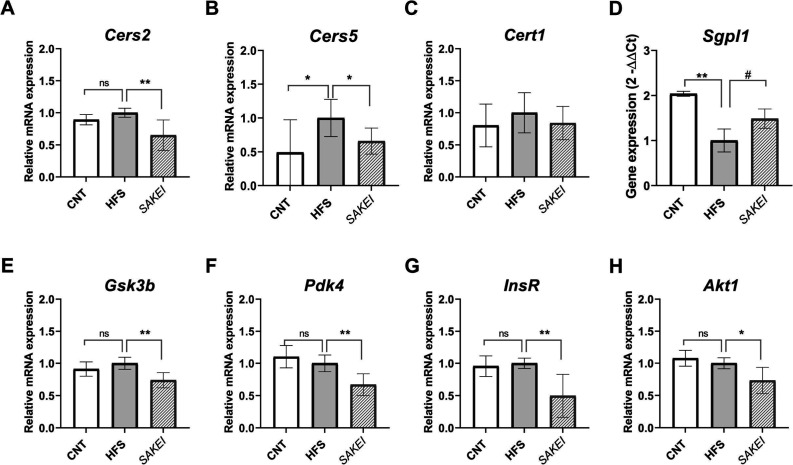
Gene expression analyses in liver samples quantified by real-time PCR (qPCR). (A) *Cers2* relative mRNA expression. (B) *Cers5* relative mRNA expression. (C) *Cert1* relative mRNA expression. (E) *Sgpl1* relative mRNA expression. (F) *Gsk3b* relative mRNA expression. (G) *Pdk4* relative mRNA expression. (H) *Insr* relative mRNA expression. (I) *Akt1* relative mRNA expression. Gene expression levels (Mean ± SEM) were normalized to the housekeeping gene (*Tbp*). Data are expressed using the 2^–ΔΔCt^ method. A one-way ANOVA followed by LSDs Fisher to evaluate statistical differences between groups (ns, not significant; # p<0.1; * p< 0.05; **p < 0.01; ***p < 0.001).

As a conclusion, the metabolic amelioration caused by *L. sakei* CNTA 173 supplementation, together with the microbiota modulation, resulted in a reduction in the sphingolipid synthesis and elongation in both WAT and liver, which promoted a lower abundance of circulating metabolites belonging to the metabolism of ceramides and sphingolipids related to the development of obesity and insulin resistance.

### 3.5 *L. sakei* CNTA 173 Does Not Induce Toxicity in Wistar Rats

In parallel to the efficacy study, we carried out an in vivo toxicological study to determine the safety of the oral administration of the probiotic *L. sakei* CNTA 173 (10^9^ CFU/animal/day) in control diet-fed rats. As shown in Figure S4, animals supplemented with the probiotic showed a slightly lower body weight at some points of the experiment, both in males and females, although these differences were not maintained at the time of sacrifice. No differences were observed in the diet intake between groups (Table S4), with slight fluctuations at some points.

After 10 weeks of administration, no lethality or alteration in the general symptoms of the animals was recorded in any of the test groups. All animals were sacrificed on day 70, according to the planned schedule. Serum biochemical (Table 2) and hematological (Table S5) determinations did not reveal suspicion of toxicity. In fact, a statistically significant decrease was detected in the serum levels of cholesterol and triglycerides of the males that received *L. sakei* 173 (*SAKEI*), as well as in the levels of the liver enzyme ALT of the females supplemented with the probiotic.

**Table 2:** Serum Biochemical Parameters of the Different Groups of the Study^a^,^b^

	males		females	
	control (*n* = 5)	SAKEI *(* *n* = 5)	control (*n* = 5)	SAKEI (*n* = 5)
albumin (g/L)	43 ± 0.2	44 ± 1	52 ± 2	50 ± 2
urea (mmol/L)	1.32 ± 0.18	1.43 ± 0.29	1.43 ± 0.18	1.25 ± 0.21
AST (U/L)	94 ± 10	89 ± 7	85 ± 14	79 ± 11
ALT (U/L)	26 ± 2	25 ± 5	22 ± 3	16 ± 4 *
ALP (U/L)	60 ± 4	61 ± 11	30 ± 6	30 ± 6
bilirubin T. (μmol/L)	1.54 ± 0. 17	1.54 ± 0.34	2.22 ± 0.51	2.40 ± 0.51
cholesterol (mmol/L)	2.64 ± 0.28	2.07 ± 0.31*	1.99 ± 0.34	2.15 ± 0.21
glucose (mmol/L)	7.27 ± 1.28	6.60 ± 0.78	6.44 ± 0.56	6.55 ± 1.05
creatinine (μmol/L)	41.55 ± 1.77	40.66 ± 2.65	47.74 ± 3.54	47.74 ± 1.77
total proteins (g/L)	66 ± 2	66 ± 1	70 ± 1	69 ± 4
triglycerides (mmol/L)	0.84 ± 0.21	0.52 ± 0.11 *	0.70 ± 0.11	0.66 ± 0.09
CPK (U/L)	714 ± 161	546 ± 164	523 ± 128	454 ± 151
LDH (U/L)	920 ± 310	707 ± 184	654 ± 178	620 ± 254

a Data correspond to the mean and SD. The statistical significance was obtained in the Mann–Whitney *U* test, according to the levels of significance: (*) significant (*p* < 0.05) and (**) very significant (*p* < 0.01).

b No differences were observed for the hematological cell counts and coagulation parameters (Tables S6 and S7, respectively).

The general external examination, as well as the macroscopic anatomopathological study of the abdominal and thoracic cavities and extracted target organs, did not show macroscopic alterations in any of the study groups. No relevant differences were observed in the weight of the different organs extracted (Tables S8 and S9) between supplemented and control animals. All findings recorded in the histological study of the selected organs: spleen, heart (atrium and ventricle), adrenal glands, lungs, liver, pancreas, kidneys, testes, ovaries, thymus, mesenteric lymph nodes, and Peyer’s patches, were considered within of the range of normal background lesions that can be observed in rats of this breed and age and under the experimental conditions used in this study. Likewise, no alterations have been found in any of the intestinal sections, small intestine (duodenum, jejunum, and ileum), and large intestine (cecum, colon, rectum)—evaluated. Consequently, under the conditions of the study, the probiotic did not cause alterations in the organs or tissues examined. Finally, no alterations in gastrointestinal symptoms or histological alterations were observed in the sections of the gastrointestinal tract evaluated: esophagus, stomach, mesenteric lymph nodes, Peyer’s patches, small intestine (duodenum, jejunum, and ileum), and large intestine (cecum, colon, and straight), indicative of good local tolerance.

## 4 Discussion

Obesity is characterized by an excessive accumulation of adipose tissue, particularly visceral WAT.^15^ This visceral fat functions as a metabolically active endocrine organ, exhibiting enhanced secretion of bioactive molecules and activation of various signaling pathways, including adipokines and cytokines, which collectively contribute to a chronic low-grade inflammatory state.^16^ Far from being merely a cosmetic concern, scientific evidence has demonstrated that this chronic low-grade inflammation of excessive adipose tissue plays a key role in the development of obesity-related metabolic complications, including insulin resistance, dyslipidemia, and MASLD.^15,16^

Different therapeutic strategies have been proposed to target visceral adiposity and mitigate the associated chronic, low-grade inflammation. These approaches encompass conventional weight-loss interventions as well as pharmacological treatments, including statins and GLP-1 receptor agonists.^17^ Nevertheless, long-term adherence to these regimens remains a significant challenge, and the incidence of adverse effects frequently compromises their effectiveness and limits the achievement of durable therapeutic outcomes.

As part of the search for novel therapeutic alternatives to counteract obesity-associated comorbidities, such as visceral fat accumulation and low-grade systemic inflammation, probiotics have gained increasing attention due to their potential modulatory effects on host metabolism and immune responses. In this sense, we had previously described the strain *L. sakei* CNTA 173 as a probiotic candidate able to significantly exert metabolic improvements in *C. elegans*, including the reduction in fat accumulation, enhancement of the oxidative stress response, and extending lifespan by directly regulating the carbohydrate and lipid metabolism.^10^ From this perspective, the present study aimed to investigate the antiobesogenic potential of this specific probiotic strain using a mammalian model of diet-induced obesity.

Regarding fat accumulation, the analyses of the individualized WAT depots demonstrated that *L. sakei* 173-treated animals showed a significant reduction of mesenteric and subcutaneous fat percentages after 10 weeks of supplementation. This result, together with previous screenings performed on *C. elegans* by our group, supports the hypothesis that *L. sakei* CNTA 173 might interfere with lipid metabolism and energy balance through a mechanism yet to be elucidated, so that the animal model accumulates a lower percentage of fat. Similar results regarding the reduction of body weight gain and fat accumulation obtained through the supplementation with *L. sakei* strains (*L. sakei* Probio65, *L. sakei* ADM14, *L. sakei* CJLS03, and *L. sakei* WIKIM31) have already been described in a similar animal model, the DIO mice.^9,18–22^ Gathering these publications, our results on *L. sakei* CNTA 173 further contribute to the establishment of the antiobesogenic properties of this species.

The gene expression analyses performed in mesenteric fat depots revealed a significant reduction in the gene expression of the *Lep* gene, coding for leptin, an adipokine that plays a crucial role in body weight regulation and lipolysis, and that has been strongly associated with obesity.^23,24^ The lower fat accumulation in subcutaneous adipose tissue has already been linked to lower leptin circulating levels.^24,25^ Thus, the reduced levels of this adipokine might be explained by the reduction in mesenteric and subcutaneous fat depots induced by the probiotic. On the other hand, even though lower circulating levels of adiponectin have been associated with obesity and insulin resistance,^26,27^ it has also been described how this could mean a turnover in the resistance to adiponectin caused by the high-fat diet, which has been described in the literature.^28–30^ This could lead to *L. sakei*-treated rats needing lower levels of circulating adiponectin to fulfill its function, and explain the reduced levels in the expression of the *Adipoq* gene induced by the probiotic. Moreover, the probiotic induced the down-regulation of adipogenesis-key transcription factors, including peroxisome proliferator-activated receptor gamma (*Pparg*), a key regulator of primary adipose tissue and an adipogenic promoter of adiponectin expression, and the Sterol regulatory element-binding protein 1 (*Srebf1*).^31–34^ Finally, SAKEI-animals showed significantly reduced levels in the expression of genes coding for the FA-binding protein 4 (*Fabp4*), also involved in the formation of mature adipocytes,^34,35^ and perilipin (*Plin*), which encodes structural proteins required for the formation of lipid droplets,^36^ in comparison with the nontreated HFS-fed rats. Interestingly, these changes were accompanied by the strong overexpression of Pgc1a, coding for a protein that enhances mitochondrial biogenesis and oxidative metabolism, promoting lipid utilization and promoting the reduction of fat accumulation in adipose tissue.^37^ The down-regulation of these genes by *L. sakei* CNTA 173 goes in line with previous works describing the ability of probiotics to modulate their expression and generate beneficial effects.^37^ The decrease in lipogenesis and a lower percentage of lipids give an insight into the possible changes that probiotics are able to induce in the expression of metabolism-related genes. In fact, further gene expression analyses demonstrated that SAKEI adipocytes showed a significant down-regulation of insulin signaling-related genes *Pdk4*, *Slc2a4,* and *Insr*. The downregulation of Pdk4 gene expression might suggests enhanced glucose oxidation and reduced triglyceride accumulation,^38^ while the reduced expression of *Slc2a4* and *Insr*, linked to the previous transcriptional changes, might suggest the remodeling of the mesenteric adipocytes induced by *L. sakei*: despite lower insulin receptor and glucose transporter mRNA levels, the reduction in lipid storage and the enhanced oxidative capacity likely promote a metabolically healthier adipose tissue, which could contribute to improved systemic insulin sensitivity.^39^ In fact, these results would be supported by the enhanced glucose tolerance previously observed in IGTT induced by the probiotic.

The lower percentage of mesenteric fat promoted by *L. sakei* CNTA 173 goes along with the literature, where the higher accumulations of fat in this metabolically active deposit have been associated with gut inflammation.^40^ Published in their work about Crohńs disease, Ha et al. discussed how gut dysbiosis could be one of the causes of hypertrophy at the mesenteric fat deposits, suggesting how certain bacteria can stimulate the inflammatory profile via M2 macrophages.^41^ This modeling of a defensive barrier that serves to prevent harmful bacteria from entering the bloodstream could theoretically be reversed by the addition of probiotics such as *L. sakei* CNTA 173, which might regulate gut microbiota and therefore lower the low-grade inflammatory profile and the excessive accumulation of mesenteric fat.

As previously mentioned, the excess of WAT characteristic of obesity contributes to the development of low-grade inflammation, which plays a crucial role in its physiopathology and acts as a link to other comorbidities. People suffering from obesity show an elevated secretion of pro-inflammatory cytokines, including TNF-α, IL-6, and MCP-1 from the hypertrophied adipose tissue, which contributes to systemic inflammation.^42^ In this regard, we evaluated circulating levels of the inflammatory markers MCP-1 and CRP-1. *L. sakei* CNTA 173 was able to significantly lower the circulating levels of CRP compared to the HFS group. No significant differences between groups were found when comparing MCP-1 plasma levels, possibly due to the high deviation within the groups. This favorable result is supported by other publications that describe the anti-inflammatory effects of other probiotics or probiotic blends. For example, the work by Webberley et al.^40^ highlighted the anti-inflammatory properties of a Lab4 probiotic blend, which improved plasma cholesterol profiles and reduced inflammatory cytokines in Wistar rats over a 90 day supplementation period.^43^

Another hallmark of obesity and other metabolic syndrome-related diseases, such as MASLD, is the dysregulation of glucose metabolism.^44^ Even though the AUC did not significantly decrease with *L. sakei* CNTA 173, the significant differences in the glycemia levels at certain time points of the IGTT suggest that this strain could have normoglycemic effects. Previous studies have related the supplementation with probiotics to better maintenance of glucose levels and insulin regulation. As previously described by Lim et al., the use of *L. sakei* OK67 induced a better response to glucose injection in DIO mice through the direct modulation of microbiota and lipopolysaccharide production, which upregulated colon tight junction protein expression.^45^ Furthermore, in the study published by Hou et al., a multistrain-probiotic mix containing *L. sakei* was shown to improve glucose tolerance and insulin sensitivity in DIO mice.^46^

The normoglycemic effect of this strain could not be confirmed in the subsequent biochemical analysis since no significant differences were observed in the glucose, insulin, and IR-HOMA and TyG indices between any of the three groups (including the control-diet group), showcasing the lack of development of a diabetic state due to the HFS diet. Furthermore, no differences were observed between any of the studied biochemical parameters besides the hepatic triglyceride content, highlighting the partial result obtained and possibly masking some of the possible probiotic effects.

The relationship between microbiota, obesity, and related conditions has been a hot topic in the last years, with studies showing correlations between certain bacteria and physiological or pathological states.^47^ The alterations in gut microbiota associated with pathological states have been defined as dysbiosis, strongly correlated with a higher incidence of complications occurring within obesity, such as type 2 diabetes.^47–49^ In this context, the use of modulatory agents of the microbiota, like probiotics, might have a crucial role in the prevention and treatment of various diseases, including obesity.^50,51^ It has been previously reported that these modulations in gut microbial composition and diversity can directly influence metabolic pathways, host immune responses, and energy homeostasis.^52^ Additionally, specific microbial taxa may mediate the probiotic’s beneficial outcomes, either by producing bioactive metabolites such as short-chain fatty acids or by modulating the gut barrier and systemic inflammation. Thus, we analyzed the fecal microbiota by 16S sequencing to elucidate possible mechanisms of action through the modulation of bacterial relative abundances and to confirm the presence of genetic material from our probiotic species. It is hypothesized that the addition of a new bacterial strain that competes for intestinal colonization will generate changes in the composition of the microbiota. Therefore, our analysis might differ from the analysis between nontreated individuals with pre-existing conditions that might have other correlations between bacterial strains or families and phenotypical factors.

Also, it must be noted that the comparisons in the gut microbiota were focused on the differences between the HFS and the HFS+ *L. sakei* CNTA 173 group, leaving out the control diet group due to substantial differences in the composition of the diet. This difference in diet composition, mainly in fat, fiber, and sugar, established a microbiota widely different from the one present in the HFS groups. The results of alpha diversity are contrary to the published bibliography, where it has been described that alpha diversity is lower in rodents fed HFS diets or prone to obesity.^50,52^ However, these results must be considered with caution, as the CNT and HFS diets differ in ingredients and macronutrient composition, and comparisons between both could lead to wrong assumptions.

The next step was to confirm the presence of the probiotic *L. sakei* CNTA 173 in the feces of the rats. Thus, this material was only detected in the treatment group, indicating the presence of the probiotic in the DIO Wistar rats and being in turn a control for the lack of cross-contamination between the HFS and the treatment groups, which received the same diet. Interestingly, the relative abundance of *L. sakei* negatively correlated with total body weight, subcutaneous fat percentage, and CRP circulating levels, highlighting its influence on the health-promoting effect previously observed. Moreover, the species *Isobaculum melis* was significantly more abundant in the probiotic group in comparison with the HFS animals. Originally isolated from the intestinal microbiota of the European badger (*Meles meles*),^53^ this Gram-positive, rod-shaped bacterium has been attributed with the capability to ferment a variety of carbohydrates, potentially contributing to the production of beneficial metabolic byproducts, such as short-chain fatty acids. Although there is no information in the literature about *I. melis* metabolism and function, the increase in the relative abundance of *I. melis* could possibly contribute to the observed beneficial effects observed by *L. sakei* administration.

Following the 16S analysis, the metabolome of the treated and HFS rats was compared. Although the low identification of metabolites significantly distributed between the groups has been an important limitation of our study, our analysis revealed a negative correlation between six potential metabolites and the abundance of *L. sakei* CNTA 173 in fecal samples. A negative correlation was also observed between some of these metabolites and the species *Isobaculim melis*, which was significantly more abundant in the probiotic group, but with lower correlation levels than those observed with *L. sakei*. Three of the metabolites, identified as Cer­(d18:0/39:0), LPIM2­(18:0/0:0), and Cer (t20:0/22:0), are all related to the sphingolipid and ceramide metabolism, which were shown to be more abundant in HFS nonsupplemented rats.

Sphingolipids are a major class of lipids composed of one polar headgroup and two nonpolar tails, sharing a common core structure based on the long-chain amino alcohol sphingosine.^54^ The metabolism of sphingolipids has been increasingly linked to obesity and its associated comorbidities, such as metabolic syndrome.^55,56^ Among them, ceramides are key components of the plasmatic membrane, which can influence the membrane properties and enzyme responses, depending on their abundance and distribution. When ceramide biosynthesis is upregulated, these molecules tend to accumulate in tissues, contributing to metabolic dysfunction.^57^ Recent research has revealed that, in obesity accompanied by insulin resistance, accumulated ceramides impair insulin signaling by inhibiting hormone-sensitive lipase, leading to increased circulating free fatty acids, a hallmark of obesity.^55,56^

Another mechanism connecting ceramide metabolism with obesity is lipotoxicity caused by excessive nutrient intake and the resulting elevated lipid levels. This process promotes ceramide synthesis, which triggers β-cell apoptosis by releasing cytochrome *c* from the mitochondria and activating the apoptotic cascade.^55,58^ Thus, lipotoxicity may drive ceramide overproduction, disrupt the liver, kidneys, and muscles function, and lead to endoplasmic reticulum (ER) stress—all key events contributing to insulin resistance and cell death, involved in obesity progression.^56^ Finally, a shift in the abundance of sphingolipids can lead to an increase in the caloric uptake through satiety-signaling and a disturbance of fat storage and adipokine secretion. Furthermore, their oxidative activity enhances oxidative stress and promotes a pro-inflammatory state, further exacerbating metabolic disturbances.^59^

Thus, the reduced circulating levels of ceramides and other sphingolipids observed in *L. sakei* CNTA 173-treated rats, compared with the HFS group, as identified by untargeted metabolomics, may be associated not only with the probiotic’s fat-reducing and antilipogenic effects, but also with the decreased CRP levels observed in DIO rats supplemented with *L. sakei* CNTA 173.

To shed light on this hypothesis, we analyzed the expression of different genes involved in ceramide metabolism to demonstrate the reduced ceramide synthesis observed in the probiotic-treated group. The gene expression analyses performed in mesenteric fat samples showed the significant down-regulation of *Cers2*, *Cers5*, *Cert1*, *Sphk1,* and *Sgpl1* genes in the SAKEI samples, in comparison with the HFS group. *Cers2* and *Cers5* encode for the ceramide synthase 2 and ceramide synthase 5, respectively, key enzymes involved in the biosynthesis of ceramides, a class of bioactive sphingolipids that regulate various cellular processes, including cell signaling, apoptosis, and lipid metabolism.^60^ *Cert1* codes for the ceramide transporter 1, a lipid transfer protein that specifically delivers ceramide from the ER to the Golgi apparatus, where ceramide serves as the substrate for sphingomyelin synthesis.^61^ In the case of sphingomyelin, the most abundant type of sphingolipid, its synthesis, and degradation are mediated by the proteins encoded by the genes *Sgpl1*, coding for the sphingosine phosphate lyase 1, and *Sphk1*, coding for the sphingosine kinase 1. Thus, their general down-regulation might indicate a significant reduction in the synthesis of sphingosine-1-phosphate (S1P), a phospholipid whose levels are associated with the development of type-2 diabetes and obesity.^62,63^ Interestingly, the simultaneous reduction of both the ceramide synthesis and the impaired production of S1P suggests that our probiotic is negatively modulating the sphingolipid pathway in WAT, which has been associated with the chronic inflammation of adipose tissue and metabolic dysfunction in obesity. The results were also supported by those observed in liver samples, where we observed the downregulation of the ceramide biosynthesis-related genes *Cers-2* and *Cers-5*, evidencing the reduction in the synthesis of these lipids in the probiotic-treated group.^64^ Moreover, *Sgpl1* showed a tendency to be upregulated in the liver samples of the SAKEI group in comparison to the untreated rats. The sphingosine phosphate lyase 1 (SGPL1) is a key enzyme responsible for the irreversible degradation of S1P in the liver.^65^ This reaction converts S1P to hexadecenal and phosphoethanolamine, representing the final step in sphingolipid metabolism. By degrading S1P, SGPL1 regulates intracellular levels of this signaling molecule, which plays crucial roles in cell proliferation, apoptosis, inflammation, and immune responses, and has been associated with obesity and insulin resistance.^65^ Finally, the reduction in ceramide synthesis and enhanced S1P degradation in the liver may be closely linked to the decreased expression of hepatic *Gsk3*, *Pdk4*, *Insr,* and *Akt1* genes, all of which play central roles in insulin signaling and glucose homeostasis. As previously mentioned, ceramides are known to interfere with insulin signaling by promoting lipotoxicity and, in turn, insulin resistance.^63,66^ Therefore, the lower ceramide content and increased S1P degradation might result in reduced activation of downstream components of the insulin pathway, as reflected by the lower hepatic expression levels of these genes. These results suggest that modulation of hepatic ceramide metabolism by *L. sakei* CNTA 173 not only affects lipid homeostasis and visceral fat distribution but also contributes to the improvement of insulin sensitivity and overall metabolic regulation, thereby contributing to the attenuation of the inflammatory state.

Finally, a parallel in vivo study was performed to demonstrate the safety of the probiotic administration, which is essential to consider the potential application of this probiotic in clinical interventions driven by metabolic syndrome. The first result of this toxicity study was the significant weight differences throughout the study between the control and *L. sakei*-treated groups, in both female and male rats. However, the analysis of the caloric intake revealed major shifts in the food consumption during those same weeks or the previous ones. Hence, the weight modulations were not considered relevant and could not be directly attributed to a metabolic shift, as described in other works.^67^. However, probiotic consumption has been shown to modulate satiety signals and hormone secretions, modifying feeding behavior.^68^ Hence, *L. sakei* CNTA 173 could have been modifying the feeding behavior and thus explaining the reduced caloric intake. However, further and longer studies should be conducted on this matter to ensure the causality of the observed effects.

Interestingly, the toxicological study allowed us to identify the significant decrease detected in the serum levels of cholesterol and triglycerides of the males that received *L. sakei* 173, as well as in the levels of the liver enzyme ALT of the females supplemented with the probiotic. This result goes in line with other studies that have shown that *Lactobacillus* species can reduce cholesterol levels.^69^ Similarly, the decrease in ALT levels in female rats supplemented with *L. sakei* CNTA 173 suggests a hepatoprotective effect, as lower ALT levels are indicative of an improved liver function. Probiotic-induced reductions in ALT have been documented in both preclinical and clinical studies.^70^ Although this effect had not been observed in the functional evaluation, it is worth mentioning that the biochemical analyses did not reveal significant changes between the HFS diet- and control diet-fed groups, demonstrating the failure in the development of the hyperglycemia and dyslipidemia characteristic of obesity in our DIO Wistar model.

Thus, the toxicological study showed how the probiotic *L. sakei* CNTA 173, dispensed together with the food for 10 weeks at a dose of 10^9^ CFU/animal/day, did not produce lethality or relevant toxic effects and is established as the dose without observed adverse effect (NOAEL). Moreover, the study further contributed to the characterization of health-benefiting effects of the probiotic, including the significant decrease of circulating cholesterol, triglycerides, and ALT levels.

Our study presents certain limitations, including the limited identification of potential differentially abundant metabolites following *L. sakei* administration as well as the lack of definitive identification through the use of standards. Further studies are warranted to clarify the exact mechanisms by which this probiotic exerts its antilipogenic activity.

However, we can conclude that the oral administration of the probiotic *Latilactobacillus sakei* CNTA 173 counteracts the obesogenic effects of a high-fat diet in obese rats, improving glucose tolerance and inflammation markers and reducing mesenteric and subcutaneous fat depots. These effects were accompanied by a probiotic-induced modulation of the ceramide and sphingolipid biosynthesis pathways, as confirmed by both by untargeted metabolomics and gene expression analyses performed in WAT and liver samples. Moreover, the supplementation induced the modulation of the gut microbiota composition, evidenced by a significant increase in the abundance of *Latilactobacillus sakei* and *Isobaculim melis* species in the feces of the treated animals, in comparison with the nontreated group. Finally, the dose of 10^9^ CFU/animal/day for *L. sakei* CNTA 173 was established as the dose without the observed adverse effect (NOAEL). Our results highlight the potential use of *Latilactobacillus sakei* CNTA 173 as a safe and effective probiotic for the prevention of the physiological and metabolic comorbidities associated with obesity and other metabolic syndrome-related diseases.

## Supplementary Material



## Notes

16S sequencing study between CNT, HFS, and *L. sakei* 173 groups, rarefaction curve representing the samples within each group, 16S sequencing study, alpha diversity comparisons among groups, Shannon (A) and Simpson (B) index analyses were performed using an ANOVA, followed by a Welch *t*-test post hoc, results are plotted as individual index values, mean ± SD, *L. sakei* 173 induces metabolic changes that correlate to its relative abundance in DIO Wistar rat fecal samples, (A) PCA of serum metabolome, both negative (top) and positive (bottom) polarities represented also in volcano plots, weight record of the body weight of the animals throughout the study, data for male rats (*n* = 5 per group), data for female rats (*n* = 5 per group), gene expression probes used for the Wistar rat quantitative real-time PCR analysis, organ and tissue weights of the control, HFS, and *L. sakei* CNTA 173-treated groups, data are expressed as the mean ± SD, biochemical parameters of the control, HFS, and *L. sakei* CNTA 173-treated groups, one-way ANOVA statistical analysis was performed, followed by uncorrected Fisher’s LSD test for multiple comparisons (**p* < 0.05), data are expressed as the mean ± SEM, daily diet consumption (g) per animal for the different groups and sex of the study, hematological parameters of the different groups of the study, data corresponding to the mean and SD, hematological cell count, data corresponds to mean (x10^3^cel/ml) and SD, coagulation parameters of the different groups of the study, data corresponding to the mean and SD, organ weight of male rats (in g) after sacrifice, data corresponding to the mean (g) and SD, organ weight of female rats (in g) after sacrifice, and data corresponding to the mean (g) and SD (PDF)

## Abbreviations

ALT: alanine aminotransferase
AST: aspartate aminotransferase
CFU: colony-forming units
CRP: C-reactive protein
DIO: diet induced obesity
DM: diabetes mellitus
FA: fatty acid
HDL: high-density lipoprotein
HFS: high-fat/high-sucrose
HOMA-IR: homeostasis model of the insulin resistance
LC-MS: liquid chromatography–mass spectrometry
MCP-1: monocyte chemotactic protein-1
MS: metabolic syndrome
MASLD: nonalcoholic fatty liver disease
IGTT: intraperitoneal glucose tolerance test
qPCR: quantitative real-time PCR
TG: triglycerides
WAT: white adipose tissue
