# Supplementary material for: Latilactobacillus sakei CNTA 173 Reduces Fat Mass by Modulating Sphingolipid Metabolism in Diet-Induced Obese Wistar Rats
Source: J Agric Food Chem. 2025 Oct 30;73(45):28848–63. doi: 10.1021/acs.jafc.5c10238 (PMC12616695; doi:10.1021/acs.jafc.5c10238)
Supplement: Supplementary file 1 [file jf5c10238_si_001.pdf]

Supporting information

Latilactobacillus sakei CNTA 173 reduces fat mass by modulating sphingolipid metabolism in diet-induced obese Wistar rats

Ignacio Goyache [1 2], Lorena Valdés-Varela [3], Raquel Virto [3], Iñigo Clemente-Larramendi [1 2], Miguel López-Yoldi [2], Ana Romo-Hualde [2], Ana Gloria Gil [4,5], Fermín I. Milagro [1,2,6,7], Paula Aranaz [2,6]*

1. University of Navarra, Faculty of Pharmacy and Nutrition, Department of Nutrition, Food Science and Physiology, 31008 Pamplona, Spain
2. University of Navarra, Center for Nutrition Research, c/Irunlarrea 1, 31008 Pamplona, Spain
3. CNTA, Ctra. NA-134 Km.53, 31570, San Adrián, Navarra, Spain
4. Department of Pharmacology and Toxicology, University of Navarra, 31008, Spain.
5. Toxicology Unit, Drug Development Unit University of Navarra (DDUNAV), University of Navarra, 31008, Spain
6. Navarra Institute for Health Research (IdiSNA), Pamplona, 31008, Spain
7. Centro de Investigación Biomédica en Red de la Fisiopatología de la Obesidad y Nutrición (CIBEROBN), Instituto de Salud Carlos III, 28029, Madrid, Spain

* Corresponding author: fmilagro@unav.es

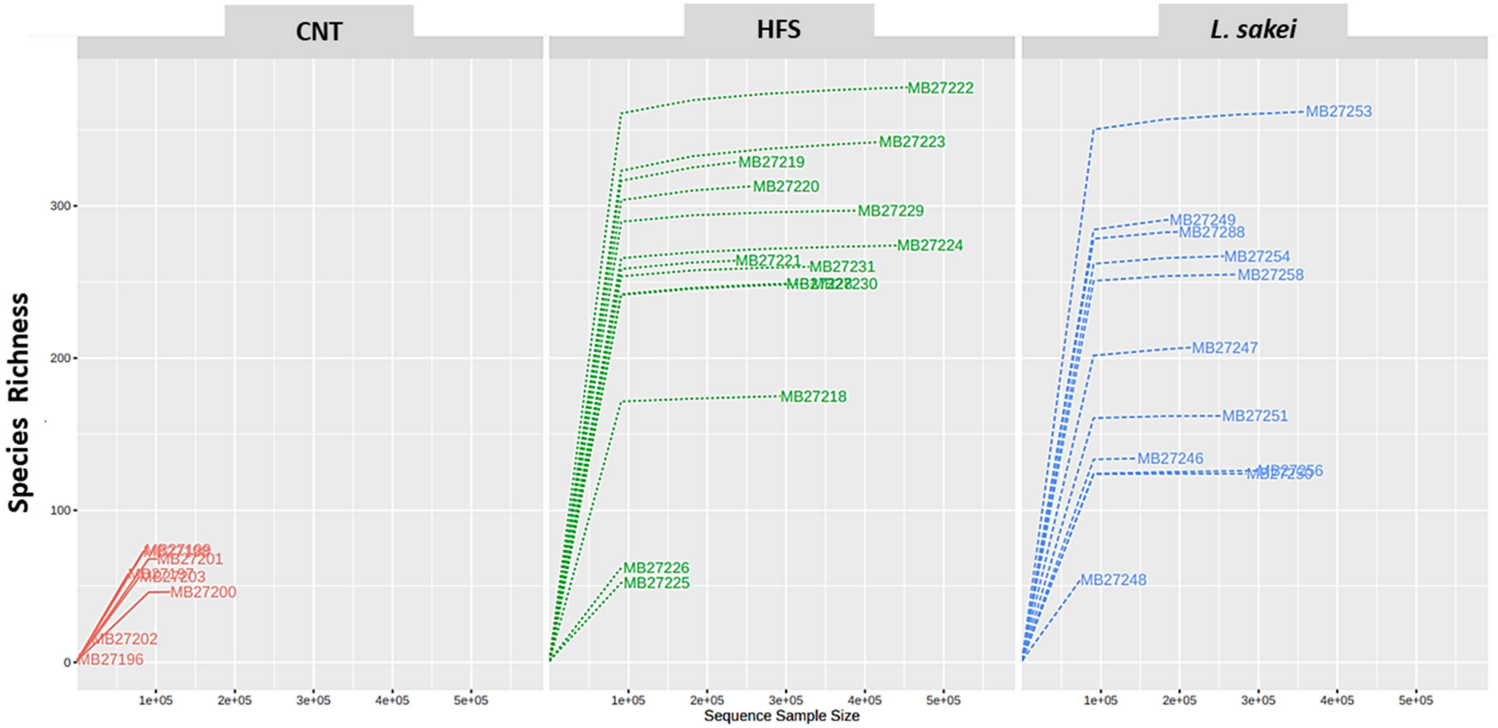

Figure S1: 16S sequencing study between CNT, HFS and SAKEI groups. Rarefaction curve representing the samples within each group.

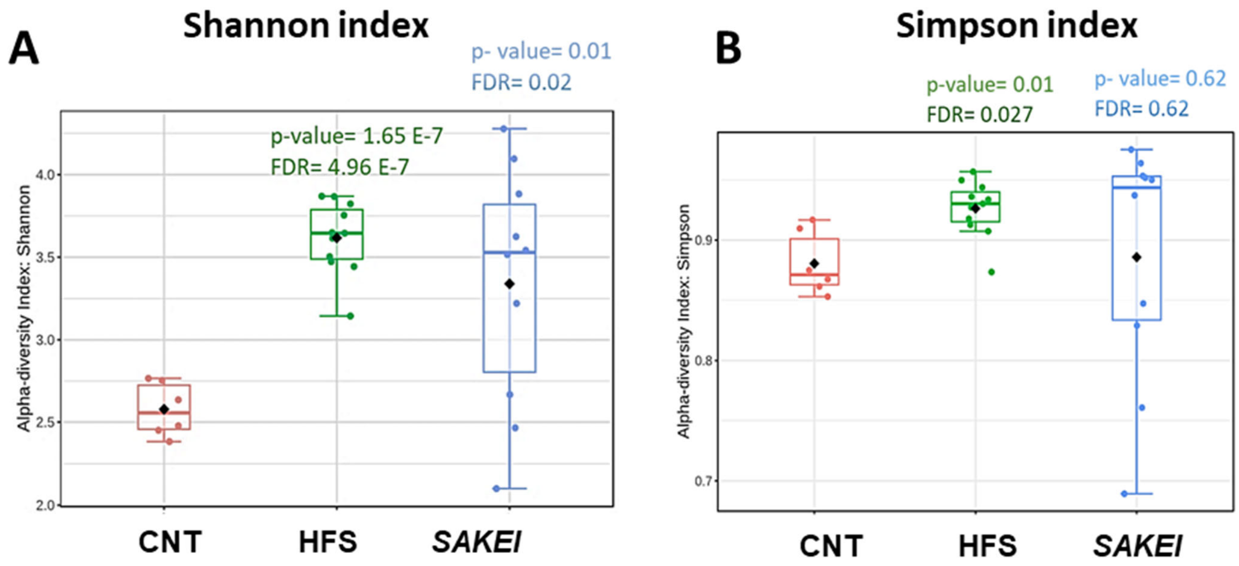

Figure S2. 16S sequencing study. Alpha diversity comparisons among groups. Shannon (A) and Simpson (B) index analyses were performed using an ANOVA followed by a Welch T-test post hoc. Results are plotted as individual index values; Mean ± standard deviation.

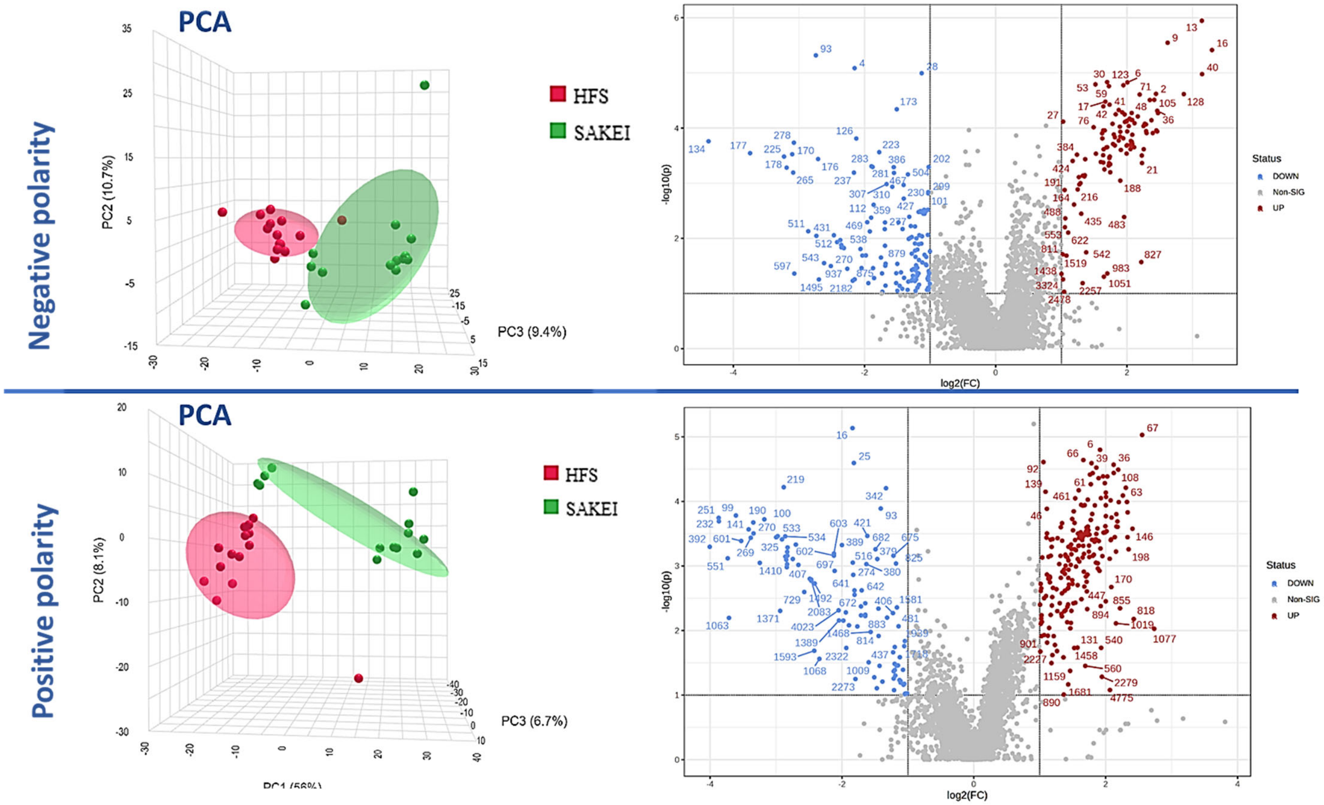

Figure S3. *L. sakei* 173 induces metabolic changes which correlate to its relative abundance in DIO Wistar rat faecal samples. (**A**) Principal component analyses (PCAs) of serum metabolome both negative (top) and positive (bottom) polarities represented also in volcano plots.

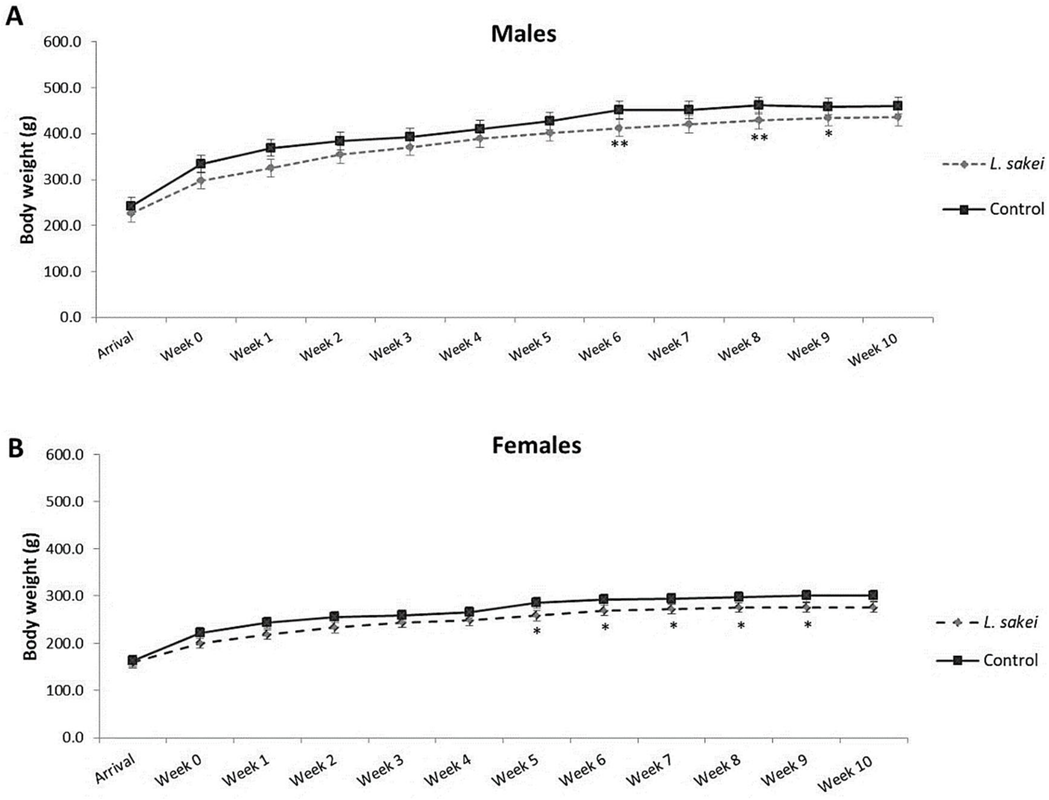

Figure S4: Weight record of the body weight of the animals throughout the study. A) Data for male rats (n=5 per group). B) Data for female rats (n=5 per group).

Supplementary tables

Table S1: Gene expression probes used for the Wistar rat quantitative real time PCR analysis.

Gene	Description	TaqMan™ Gene Expression Assay ID
Acox1	Acyl-Coenzyme A oxidase 1, palmitoyl	Rn01460628_m1
Acot8	Acyl-CoA thioesterase 8	Rn00590721_m1
Adipoq	Adiponectin, C1Q and collagen domain containing	Rn00595250_m1
Fabp4	fatty acid binding protein 4	Rn00670361_m1
Hsd17b4	Hydroxysteroid (17-beta) dehydrogenase 4	Rn00577789_m1
Lep	Leptin	Rn00565158_m1
Plin1	perilipin 1	Rn00558672_m1
Pparg	Peroxisome proliferator activated receptor gamma	Rn00440945_m1
Scp2	Sterol carrier protein 2	Rn00684513_m1
Srebf1	Sterol regulatory element binding transcription factor 1	Rn01495769_m1
Tbp	TATA box binding protein. Housekeeping gene control	Rn01455646_m1

Table S2: Organ and tissue weights of the control, HFS and *L. sakei* CNTA 173-treated groups. Data are expressed as the mean ± SD.

	CNT (*n* =8)	HFS (*n* =14)	*SAKEI* (*n* = 14)
**Liver**	10.43 ± 0.91	10.36 ± 0.96	10.36 ± 0.93
**Spleen**	0.70 ± 0.09	0.93 ± 0.23	0.86 ± 0.25
**Kidney**	1.19 ± 0.10	1.24 ± 0.10	1.23 ± 0.12

Table S3: Biochemical parameters of the control, HFS and *L. sakei* CNTA 173-treated groups. One-way ANOVA statistical analysis was performed followed by uncorrected Fisher´s LSD test for multiple comparisons (* $p < 0.05$). Data are expressed as the mean ± SEM.

	CNT (*n* =8)	HFS (*n* =14)	*SAKEI* (*n* = 14)
**Glucose (mmolL^{-1})**	6.15 ± 0.22	6.32 ± 0.17	6.34 ± 0.19
**Insulin (mU L^{-1})**	0.06 ± 0.01	0.10 ± 0.05	0.10 ± 0.02
**Cholesterol (mmolL^{-1})**	2.58 ± 0.15	2.41 ± 0.12	2.32 ± 0.12
**HDL (mmolL^{-1})**	0.65 ± 0.05 *	0.56 ± 0.02	0.54 ± 0.02
**Triglycerides (mmolL^{-1})**	1.47 ± 0.19	1.37 ± 0.09	1.30 ± 0.18
**ALT (U L^{-1})**	49.5 ± 4.4	43.9 ± 2.3	43.4 ± 3.5
**AST (U L^{-1})**	144.1 ± 13.8	161.6 ± 14.9	171.4 ± 18.4

Table S4: Daily diet consumption (g) per animal for the different groups and sex of the study.

Week	MALES		FEMALES	
	**Control** (n=5)	***SAKEI*** (n=5)	**Control** (n=5)	***SAKEI*** (n=5)
Arrival	21.8	21.8	16.2	16.2
Week 1	24.7	23.8	17.7	17.1
Week 2	29.2	23.5	19.2	16.8
Week 3	29.1	23.9	19.1	16.7
Week 4	28.8	24.3	19.3	17.2
Week 5	28.9	23.6	18.7	17.0
Week 6	24.6	24.6	19.2	17.8
Week 7	29.0	23.9	18.9	17.6
Week 8	28.9	24.3	19.0	17.7
Week 9	28.5	23.6	19.3	17.6
Week 10	29.6	24.4	19.6	17.4

Table S5: Hematological parameters of the different groups of the study. Data corresponds to the mean and standard deviation.

	MALES		FEMALES	
	**Control** (n=5)	***SAKEI*** (n=5)	**Control** (n=5)	***SAKEI*** (n=5)
White blood cells ($x10^3$cel/ml)	6.31 ± 0.97	5.24 ± 1.29	3.41 ± 0.81	2.83 ± 0.46
Red blood cells ($x10^6$cel/ml)	9.44 ± 0.37	9.64 ± 0.42	8.05 ± 0.25	8.22 ± 0.21
Hemoglobin (g/dl)	15.6 ± 0.5	15.8 ± 0.3	14.6 ± 0.4	14.4 ± 0.6
Hematocrit (%)	47.4 ± 1.3	47.6 ± 0.8	43.3 ± 1.4	43.4 ± 1.5
Mean corpuscular volume (fl)	50.3 ± 2.2	49.4 ± 1.8	53.9 ± 2.0	52.8 ± 1.0
Mean corpuscular hemoglobin (pg)	16.5 ± 0.7	16.4 ± 0.6	18.2 ± 0.7	17.6 ± 0.4
Mean corpuscular hemoglobin concentration (g/dl)	32.9 ± 0.2	33.3 ± 0.2 *	33.7 ± 0.1	33.3 ± 0.2
Platelets ($x10^3$cel/ml)	804 ± 110	780 ± 60	757 ± 63	717 ± 60
Reticulocytes (%)	2.71 ± 0.3	3.08 ± 0.26	2.43 ± 0.44	2.65 ± 0.56

The statistical significance was obtained in the Mann Whitney U test, according to the levels of significance: (*) significant ($p < 0.05$).

Table S6: Hematological cell count. Data corresponds to the mean ($x10^3$cel/ml) and standard deviation.

	MALES		FEMALES	
	**Control** (n=5)	***SAKEI*** (n=5)	**Control** (n=5)	***SAKEI*** (n=5)
Neutrophils	1.36 ± 0.61	1.28 ± 0.55	0.59 ± 0.14	0.46 ± 0.14
Lymphocytes	4.31 ± 0.70	3.54 ± 1.04	2.62 ± 0.81	2.23 ± 0.48
Monocytes	0.35 ± 0.13	0.27 ± 0.18	0.09 ± 0.02	0.08 ± 0.02
Eosinophils	0.09 ± 0.03	0.06 ± 0.02	0.05 ± 0.02	0.04 ± 0.01
Basophiles	0.19 ± 0.12	0.09 ± 0.04	0.05 ± 0.02	0.03 ± 0.01

Table S7: Coagulation parameters of the different groups of the study. Data corresponds to the mean and standard deviation.

	MALES		FEMALES	
	**Control** (n=5)	***SAKEI*** (n=5)	**Control** (n=5)	***SAKEI*** (n=5)
Fibrinogen (mg/dL)	253.1 ± 31.4	238.9 ± 29.9	147.5 ± 8.2	145.2 ± 13.1
PT (Prothrombin time. s)	16.0 ± 0.6	17.3 ± 1.6 *	16.4 ± 1.1	16.8 ± 1.3 *
aPTT (Thromboplastin time. s)	30.7 ± 3.9	26.7 ± 4.0	30.8 ± 1.4	27.6 ± 3.8*

The statistical significance was obtained in the Mann Whitney U test, according to the levels of significance: (*) significant (p <0.05).

Table S8: Organ weight of male rats (in g) after sacrifice. Data corresponds to the mean (g) and standard deviation.

	**Control** (n=5)	***SAKEI*** (n=5)
Spleen	0.694 ± 0.067	0.617 ± 0.052 *
Heart	1.180 ± 0.121	1.176 ± 0.115
Liver	12.287 ± 0.701	11.295 ± 0.572
Thymus	0.510 ± 0.120	0.457 ± 0.055
Right kidney	1.186[#] ± 0.093	1.246 ± 0.066
Left kidney	1.186[#] ± 0.068	1.237 ± 0.090
Kidneys (sum)	1.898[#] ± 1.067	2.484 ± 0.148
Right testis	1.938 ± 0.068	1.771 ± 0.187
Left testis	1.975 ± 0.071	1.766 ± 0.161
Testis (sum)	3.913 ± 0.128	3.537 ± 0.335
Right adrenal gland	0.045 ± 0.013	0.050 ± 0.006
Left adrenal gland	0.050 ± 0.007	0.052 ± 0.008
Adrenal glands (sum)	0.095 ± 0.020	0.102 ± 0.014

The statistical significance was obtained in the Mann Whitney U test, according to the levels of significance: (*) significant (p <0.05).

Table S9: Organ weight of female rats (in g) after sacrifice. Data corresponds to the mean (g) and standard deviation.

	**Control** (n=5)	***SAKEI*** (n=5)
Spleen	0.511 ± 0.080	0.438 ± 0.036
Heart	0.900 ± 0.047	0.772 ± 0.067 *
Liver	8.068 ± 0.931	6.971 ± 0.598
Thymus	0.342 ± 0.046	0.375 ± 0.049
Right kidney	0.913 ± 0.147	0.826 ± 0.077
Left kidney	0.899 ± 0.087	0.810 ± 0.068
Kidneys (sum)	1.812 ± 0.231	1.636 ± 0.144
Right ovary	0.066 ± 0.024	0.038 ± 0.006
Left ovary	0.069 ± 0.023	0.034 ± 0.009
Ovaries (sum)	0.135 ± 0.029	0.072 ± 0.014 *
Right adrenal gland	0.038 ± 0.005	0.040 ± 0.007
Left adrenal gland	0.044 ± 0.005	0.041 ± 0.005
Adrenal glands (sum)	0.082 ± 0.009	0.081 ± 0.012

The statistical significance was obtained in the Mann Whitney U test, according to the levels of significance: (*) significant ($p < 0.05$).
